# Comparisons of core component delivery in cardiac rehabilitation programs by country income classification and decade based on the 2025 Global Audit Update: A survey study

**DOI:** 10.1371/journal.pmed.1005151

**Published:** 2026-06-23

**Authors:** Gabriela Lima de Melo Ghisi, Rachael P. Carson, Karam Turk Adawi, Rongjing Ding, Warner M. Mampuya, Mariya P. Jiandani, Jimena Martinez, Monserrat Cruz Rivero, Claudia V. Anchique, Dinah L. van Schalkwijk, Jonathan Gallagher, Buket Akinci, Dion Candelaria, Jirapa Champaiboon, Daniel F. Quesada-Chaves, Tone M. Norekvål, Iwona Szadkowska, Borut Jug, Evangelia Kouidi, Marta Supervia, Won-Seok Kim, Chamila Mettananda, Lilian Mbau, Gulsim T. Aimakova, Sherry L. Grace, on behalf of the ICCPR Global Cardiac Rehabilitation Audit Update Investigators

**Affiliations:** 1 KITE Research Institute, University Health Network, University of Toronto, Toronto, Canada; 2 Department of Physical Therapy, University of Toronto, Toronto, Canada; 3 Faculty of Health, York University, Toronto, Canada; 4 Department of Public Health, College of Health Science, QU Health, Qatar University, Doha, Qatar; 5 Cardiac Rehabilitation Center, Peking United Medical College Hospital, Beijing, China; 6 Department of Medicine, Faculty of Medicine and Health Sciences, Université de Sherbrooke, Sherbrooke, Canada; 7 Department of Physical Therapy, Seth GS Medical College and KEM Hospital, Mumbai, India; 8 Instituto Medico DAMIC, Córdoba, Argentina; 9 Casa Corazón Otoch Puksikal, Mérida, Mexico; 10 Mediagnóstica Tecmedi SAS, Duitama, Colombia; 11 Department of Cardiology, Radboud University Medical Center, Nijmegen, The Netherlands; 12 Department of Medical and Clinical Psychology, Tilburg University, Tilburg, The Netherlands; 13 Department of Cardiology (Cardiac Rehabilitation), Beaumont Hospital, Dublin, Ireland; 14 Physiotherapy and Rehabilitation Department, Faculty of Health Sciences, Biruni University, Istanbul, Turkey; 15 Biruni University Research Center, Biruni University, Istanbul, Turkey; 16 Faculty of Medicine and Health, Susan Wakil School of Nursing and Midwifery, The University of Sydney, Sydney, Australia; 17 Charles Perkins Centre, The University of Sydney, Sydney, Australia; 18 Department of Rehabilitation Medicine, Faculty of Medicine, Chulalongkorn University, Bangkok, Thailand; 19 Hospital San Vicente de Paul, Heredia, Costa Rica; 20 Department of Heart Disease, Haukeland University Hospital, Bergen, Norway; 21 Faculty of Health and Social Sciences, Western Norway University of Applied Sciences, Bergen, Norway; 22 Department of Clinical Science, University of Bergen, Bergen, Norway; 23 Department of Sports Medicine, Medical University of Lodz, Lodz, Poland; 24 Centre for Preventive Cardiology, Department of Vascular Medicine, University Medical Centre Ljubljana, Ljubljana, Slovenia; 25 Department of Internal Medicine, University of Ljubljana, Ljubljana, Slovenia; 26 Laboratory of Sports Medicine, Faculty of Physical Education and Sport Sciences, Aristotle University of Thessaloniki, Thessaloniki, Greece; 27 Department of Physical Medicine and Rehabilitation, Gregorio Marañón General University Hospital, Gregorio Marañón Health Research Institute, Madrid, Spain; 28 Radiology, Rehabilitation and Physiotherapy Department, Complutense University School of Medicine, Madrid, Spain; 29 Division of Preventive Cardiology, Department of Cardiovascular Medicine, Mayo Clinic, Rochester, Minnesota, United States of America; 30 Department of Rehabilitation Medicine, Seoul National University College of Medicine, Seoul National University Bundang Hospital, Seongnam-si, South Korea; 31 Gyeonggi Regional Cardiocerebrovascular Disease Center, Seoul National University Bundang Hospital, Seongnam-si, South Korea; 32 Department of Pharmacology, University of Kelaniya, Faculty of Medicine, Ragama, Sri Lanka; 33 Cardiac Rehabilitation, Centre for Cardiovascular Prevention and Rehabilitation, Nairobi, Kenya; 34 UMC Heart Centre, Astana, Kazakhstan; 35 KITE—Toronto Rehab & Peter Munk Cardiac Centre, University Health Network, University of Toronto, Toronto, Canada; Simon Fraser University, CANADA

## Abstract

**Background:**

Cardiovascular disease (CVD) remains a leading global health burden. Cardiac rehabilitation (CR) is essential to reducing morbidity and improving patient outcomes. Since the COVID-19 pandemic, CR delivery worldwide has evolved, yet these changes have not been systematically charactemkjrized. The objective of this study was to characterize globally: (1) the delivery of core CR components, including risk factors assessed, patient education practices, and program resources; (2) differences in these elements by country income classification and relative to the initial 2016 Global CR Audit.

**Methods and findings:**

A cross-sectional Audit update was conducted. Program-level data were collected from May 1st to September 1st 2025 using a REDCap survey adapted from previous Audits. Eligible respondents were leads of phase II/post-discharge CR programs providing at least an initial assessment, structured aerobic exercise, and ≥1 additional core component. ICCPR associations and local leaders supported program identification. Main outcomes were core components delivered (10 assessed), risk factors assessed (14 assessed), patient education dose (hours/patient/program), and program resources (17 assessed). Generalized linear mixed models (GLMM) tested differences by income classification and (when applicable) changes since 2016. Of 7,025 programs identified globally, 1,505 (62% median country response rate) initiated a survey from 90/113 (80%) countries with CR. The median number of core components offered was 8/program (p25, p75 = 6, 10), with upper-middle income countries offering significantly more components overall (median = 9), and also high-income countries offering more than low-income countries (8 versus 6, *p* < 0.001; decade change not tested). Programs assessed 11 risk factors/program (median; p25, p75 = 8, 12). This significantly differed by country income class (GLMM *p* < 0.001), with programs in lower-middle income countries assessing fewer risk factors than those in both upper-middle-income (mean difference = 2.2; *p* < 0.001) and high-income countries (mean difference = 1.6, *p* < 0.001). There were significant increases in 2025 for glucose, sleep apnea and sedentariness, among others (ps < 0.01). Patient education dose was 3 hours/supervised program (median; p25, p75 = 1, 7), a significant reduction in many high-income countries since 2016 (*p* = 0.01). Globally, gym space, resistance training equipment, and individual assessment/counseling space were the most common resources (all >90%; median = 11; p25, p75 = 8, 14). Resource availability differed significantly by country income class (GLMM *p* < 0.001), with programs in upper-middle-income countries reporting more resources than those in high-income (mean difference = 1.5), lower-middle-income (mean difference = 2.6), and low-income countries (mean difference = 4.8; all *p* < 0.001). While there were no significant differences in total resources, resistance training equipment, electronic patient charts, body composition analyzers, and stress testing with O_2_ were more available in 2025, and the availability of administrative office space and group education room less so (ps < .01). Limitations include potential selection and ascertainment bias from incomplete program identification as well as variable, modest program response rates, limited representation from low-income settings, reliance on self-reported survey data, as well as measurement differences across Audit cycles, which may affect generalizability and precision of findings.

**Conclusions:**

CR programs worldwide continue to deliver guideline-concordant care, with education potentially shifting modality. However, modest inequities persist for resource-constrained programs.

## Author summary

**Why was this study done?:**

- Cardiac rehabilitation is a proven program that helps people recover after heart disease, but access and delivery vary widely around the world, and little is known about how consistently key parts of these programs are currently provided globally.
- The last global assessment of cardiac rehabilitation programs was conducted nearly a decade ago, before major disruptions to health systems caused by the COVID-19 pandemic and the expansion of digital care models.
- Updated global data were needed to understand whether programs are delivering recommended components of care and whether differences exist between countries with different resource levels.

**What did the researchers do and find?:**

- We conducted a global survey of 1,505 cardiac rehabilitation programs in 90 countries, representing about 80% of countries worldwide that have such programs, to assess delivery of recommended care components, patient education, risk factor assessment, and program resources.
- Most programs delivered the majority of recommended components (median 8 of 10 components globally), and nearly all programs provided patient education, although the median amount of education decreased from 4.5 hours in 2016 to 3.0 hours in 2025.
- Programs in higher- and upper-middle-income countries assessed more cardiovascular risk factors and had more resources available than those in lower-resource settings, although programs worldwide generally continued to provide comprehensive services despite constraints.

**What do these findings mean?:**

- These results show that cardiac rehabilitation programs globally continue to deliver most recommended components of care, demonstrating strong commitment to secondary prevention across diverse health systems.
- However, differences between countries by income level and reductions in reported patient education time highlight opportunities to strengthen program resources and ensure equitable access to comprehensive care.
- Because findings were based on survey responses and some countries (especially those in lower-income settings) had fewer participating programs, results should be interpreted cautiously, but they provide the most current global picture available to guide practice, policy, and future research.

## Abstract 3

In this survey study, Gabriela Ghisi and colleagues compared delivery of cardiac rehabilitation programs worldwide, comparing countries as well as comparing the data to the previous round of the ICCPR global audit.

## Introduction

The increasing incidence and reduced case fatality of cardiovascular diseases (CVD) have resulted in many people globally living chronically with—and hence requiring secondary prevention for—this leading burden of disease to optimize quantity and quality of life [1,2]. The societal, health system, and economic burden in low- and middle-income countries (LMICs) is particularly high [3].

Cardiac rehabilitation (CR) is a multi-component secondary prevention model of care delivered by a multidisciplinary team [4]. It is proven to reduce cardiovascular mortality, hospital readmissions, as well as improve the quality of life for patients with CVD [5]. However, there is variability in the implementation of CR globally [6,7], and it remains under-investigated in LMICs [8,9] where programs are particularly under-resourced despite great need [10].

Many CR Societies globally—including the American Association of Cardiovascular and Pulmonary Rehabilitation (AACVPR) [11], the European Association of Preventive Cardiology (EAPC) [12], the British Association for Cardiovascular Prevention and Rehabilitation (BACPR) [13], and the International Council of Cardiovascular Prevention and Rehabilitation (ICCPR) [4]—have delineated consistent core components to be delivered by CR programs. These core components typically include initial patient assessment with a focus on risk factors, structured exercise training, patient education, cardiovascular risk factor management, and psychosocial support [4]. These Societies also specify standards for risk factor assessment [14]. Despite these clear recommendations, variability exists in the implementation of these components worldwide [15].

Indeed, in 2016, the ICCPR conducted the first Global Audit of CR programs [16], detailing this variation [17]. Differences in program resource availability to deliver these components and assess CV risk factors were also characterized. Since then, a rapid Audit Update during the COVID-19 pandemic highlighted program closures and major changes in CR delivery among programs that remained open, including the ways in which core components and patient education were provided [18]. With program closures, it is likely availability of program infrastructure and digital resources has changed dramatically, also impacting capacity to fulsomely assess all risk factors recommended.

To understand how CR has evolved in the aftermath of the pandemic [18], ICCPR recently undertook a third Audit Update. The aims of this study were to firstly characterize the delivery of core CR components globally, including (a) risk factors assessed during initial patient assessment, (b) patient education practices (in supervised and unsupervised models), and (c) program resources to deliver these components, among other program elements and best practices (notably quality assessment and program certification). Second, these were compared by country income classification and Audit decade.

## Methods

This Global Audit Update employed a cross-sectional, observational design. Ethics approval was obtained from the York University Research Ethics Board (Toronto, Canada; e2025-057). The study protocol was approved by the ICCPR Executive Board and publicly posted prior to study initiation (S1 Protocol). Findings related to CR program staffing, accepted indications, and dose [19] are reported elsewhere; capacity, access, and referrals as well as full details regarding hybrid models will be reported elsewhere.

### Procedure

The World Health Organization’s country list served as the basis for this Audit; for the purposes of this paper, the five countries of the United Kingdom (UK) were considered separately (including Bermuda). To identify countries with existing CR programs, data on program availability from previous Audits (i.e., 111/203 countries) [16,17] were first considered. To verify any national phase II CR service provision, CR and related Societies (including ICCPR members), or other national leaders identified through peer-reviewed publications, ICCPR’s network including umbrella Associations (e.g., International Society of Physical Medicine and Rehabilitation), or organizational websites were engaged. Where any CR availability was confirmed, the number of programs meeting Audit inclusion/exclusion criteria was sought from the contact. In countries without CR, regional leads confirmed its current absence, or a local contact was made if disconfirmed.

Identified leaders in countries with ≤9 CR programs were asked to share the contact email for each program in return for free ICCPR CR Foundations Certification (CRFC; value $125USD) [20]. Identified leaders in countries with ≥10 CR programs were asked to obtain and share the program email addresses, or could elect to distribute the survey invitation letter and link through their Society program email distribution list. Those who contributed were invited to collaborate to ensure local rigor and fulsome dissemination of the results. Societies were offered a personalized national comparative results summary.

An email invitation to complete the online survey was sent to each CR program email via REDCap (SurveyMonkey in China), or by the Society leader to their distribution lists as applicable. The email requested the “lead clinician” of each program complete the survey, and that program staff communicate to ensure only one survey be completed per program. Audit participation was voluntary, and electronic informed consent was obtained prior to survey initiation. Respondents were not asked to identify themselves or their programs to reduce the potential for socially desirable responding. Participants were offered free ICCPR CRFC online training [20].

Data were collected between May 1st and September 1st, 2025. Up to two reminders were issued to non-responders at a two-week interval. ICCPR also distributed the survey invitation through their four social media channels, as well as their program and Council member email distribution lists.

### Participants

The study population comprised all Phase II CR programs identified worldwide. The study sample included all programs from the population that were successfully identified and contacted to participate in the Audit. The study survey email requested the “lead clinician” of each program complete the survey (i.e., program respondent), and that program staff communicate to ensure only one survey be completed per program.

The inclusion criteria was programs offering post-discharge services delivering at least: (1) initial patient assessment (detailed risk factors not specified); (2) structured aerobic exercise training, whether supervised or unsupervised (+/- technology); and (3) at ≥1 additional core component aimed at controlling cardiovascular risk factors (e.g., patient education, psychosocial or dietary counseling, tobacco cessation) [4]. Programs that operated solely and temporarily as part of a research study were excluded.

The setting could be residential in the case of “spa” programs, but would be primarily outpatient. Program staff were invited to contact the study email address where they were unsure about meeting these criteria, and some did. Study materials made it explicit to potential participants that Phase I or maintenance programs were not to be reported.

It is important to note that all programs were required to offer an initial assessment and structured aerobic exercise; however, supervision of exercise was not universally required. Consequently, when interpreting the prevalence of these elements across programs, caution is warranted to avoid overestimating how universally they are implemented, as these elements were part of the inclusion criteria for program participation in the survey.

### Measures

The survey instrument was based on the 2016 Global CR Audit which comprised items harmonized across available national CR program surveys as described elsewhere [16,21], and some adaptations from the COVID-19 Rapid Audit survey [18]. The survey was updated for currency, brevity, and clarity. It was then publicly posted via ICCPR for three weeks to solicit input from the global CR community; edits were made accordingly. The full survey is available in S1 Appendix. When comparable survey items were available, data from the 2016 Audit were retrieved to examine decade-level changes in CR program delivery [17,22,23].

Initial items characterized respondent and program characteristics to describe the sample. Country of program was queried. There was a section on center-based models, as well as alternative models/settings, such as home-based (+/− digital technology) and hybrid programs still meeting inclusion criteria.

Similar to the previous Audits [17,18], response options included numbers (constrained to plausible maximum and minimums to reduce human errors), closed-ended options as well as open-ended text (e.g., for “other” responses). Branching logic was applied to minimize respondent burden where applicable.

Respondents were invited to use browser-embedded software to translate the surveys using their native language where desired. To maximize response rate and comparability of items, the survey was made available in Simplified Chinese for respondents who requested it.

Additional items assessed education in home-based or hybrid program models, such as whether this component was reimbursable by a non-patient funding source, and where available, if participants in these models were provided supporting educational materials (yes/no).

This study focused on 2025 Audit items pertaining to the delivery of core CR components, risk factors assessed, patient education practices, and program resources. A total of 26 elements (including core components) were listed, and respondents were asked to indicate whether the program offered each (yes/no), or if patients were referred elsewhere to receive it. Response options were based on core components recommended by major CR Societies [15]. In some cases, the elements pertaining to a core component were grouped. Total components offered were computed.

Respondent program staff were also asked whether they assessed each of 13 risk factors (yes/no); they could also report “other” risk factors assessed open-ended. These too were also summed. With regard to patient education, program respondents were asked to report the number of formal group or one-on-one education sessions offered to each patient in their standard prescribed supervised program, as well as the average number minutes duration of each; the product was computed to characterize supervised education dose (hours/patient/program).

Another item assessed availability of 16 potential program resources (e.g., education space, equipment) to deliver the core components. Response options for each were whether the particular resource was not available, or if it was, whether access was shared or the resource was dedicated for use by their program. Finally, program respondents were asked to report if they assess program quality, as well as about their interest in program certification and CR registry participation.

### Data analyses

Data were analyzed using SPSS version 31 [24]. All initiated surveys were included in the analysis. Country information was missing for 23 (1.5%) initiated surveys; country response rate was computed based on available responses. Country of program was coded into income classifications based on the World Bank [25]. Non-parametric tests were used where data were not normally distributed.

Data were stratified by country income classification [25], and inferential tests were performed to test objectives. Clustering of program-level data within countries was addressed using generalized linear mixed models (GLMM), specifying country as a random effect. Count variables (e.g., total number of core components delivered or patient education hours) were modeled using negative binomial distributions, given non-normality of distribution of count variables assessed visually using histograms and *Q*–*Q* plots. Model residuals and fit statistics were examined to verify assumptions. Where significant, post-hoc pairwise Bonferroni comparisons were performed by income classes. Statistical significance was set at *p* < 0.05 throughout; however, where multiple comparisons were performed (e.g., comparing individual program resources by income class and decade), a more conservative threshold of *p* < 0.01 was applied, approximating a Bonferroni-style adjustment for multiple comparisons to reduce the risk of type I error, for consistency of inference across analyses.

Where comparable survey items were available, 2025 Audit data were compared with 2016 findings [17]. Where applicable, inferential tests were performed, including the chi-squared test for categorical variables and the Mann–Whitney *U* and Kruskal–Wallis tests for continuous variables.

Descriptive statistics, including frequencies and valid percentages for categorical variables, and medians with interquartile ranges (25th–75th percentiles) for non-normally distributed continuous variables were calculated using all available data, accounting for branching logic. Descriptive analyses were complemented by a heat map illustrating values by country income class and a stacked bubble chart, both created using Microsoft Excel. Open-ended responses were coded using content analysis [26].

### Use of artificial intelligence (AI)

Generative AI (ChatGPT) was used for editing assistance (language editing, improving readability), with output evaluated for validity by the senior author. The authors retain full responsibility for the content of the manuscript.

## Results

### Audit cohort

**Fig 1:**
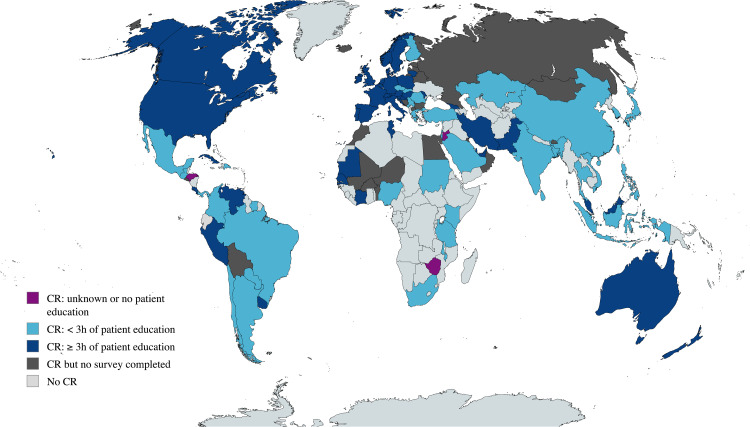
Availability of phase II cardiac rehabilitation and patient education dose/complete supervised program (hours) by country in Audit Update (2025), *N* = 199. Basemap obtained from Natural Earth (public domain): https://www.naturalearthdata.com/. Acronym: CR, cardiac rehabilitation.

As detailed elsewhere [19], 113 of 199 countries (56.8%) were confirmed to have at least one existing Phase II CR program (i.e., availability; countries without CR depicted in Fig 1), with 47 (87.0% of 54) high-income countries, 39 (54.2%/72) upper-middle-income countries, 21 (40.4%/52) lower-middle-income countries, and 6 (30.0%/20) low-income countries offering any CR services (note Venezuela could not be categorized) [25]. At least one survey was initiated by a program from 90/113 (79.6% country response rate) of these countries with any CR (see Fig 1; note in 18 [1.2%] surveys, country was not available, but responses were included in global computations to enhance generalizability).

Globally, 7,025 CR programs were identified, of which 1,505 (21.4% program response rate globally) initiated a survey (Table 1). Median program response rate by country (Table 1) was 62.3% (p25, p75 = 24.1, 100.0%). The number of responses per country are shown in Table 1; the number and proportion of responses by country income classification are shown in Table 2. The number of responses per country for the initial 2016 Audit are also shown in Table 1; response rates by country [17] and income class [25] for that decade are reported elsewhere.

**Table 1:** Education component dose^a^ in phase II cardiac rehabilitation in countries with any programs (*N* = 113) by World Bank country income classification [25], audit decade, and country.

World Bank Country Income Classification (program response rate) Country (2,016 n; 2,025 n, with program response rates)	Number of education sessions/program (mean ± SD)		Duration of education sessions minutes/session (mean ± SD)		Total Education Dose hours/program/patient (median [p25, p75])^b^	
	Global 2016 *N* = 1,082	Global 2025 *N* = 1,505	Global 2016 *N* = 1,082	Global 2025 *N* = 1,505	Global 2016 *N* = 1,082	Global 2025 *N* = 1,505
**High income (13.3%)**	**9.4 ± 12.0**	**7.8 ± 8.5**	**51.6 ± 30.6**	**48.7 ± 24.2**	**6.0 (2.6, 9.0)**	3.0 (2.0, 8.0)*
Australia (*n* = 85; *n* = 96, 24.2%)	7.8 ± 3.6	8.0 ± 4.2	54.2 ± 15.0	54.1 ± 17.5	6.8 (5.8, 9.0)	7.0 (5.0, 9.0)
Austria (*n* = 5; *n* = 3; 12.0%)	36.8 ± 62.2	SCS	68.8 ± 44.2	SCS	9.0 (3.9, 84.6)	SCS
Bahrain (*n* = 1; *n* = 1, 50.0%)	SP	SP	SP	SP	SP	SP
Barbados (*n* = 1; *n* = 1, 100.0%)	SP	SP	SP	SP	SP	SP
Belgium (*n* = 9; *n* = 3, 5.0%)	10.7 ± 15.3	SCS	98.6 ± 28.5	SCS	12.0 (3.0, 16.0)	SCS
Bermuda (*n* = 1; *n* = 1, 100.0%)	SP	SP	SP	SP	SP	SP
Brunei Darussalam (*n* = 2; *n* = 2, 100.0%)	SCS	SCS	SCS	SCS	SCS	SCS
Canada (*n* = 57; *n* = 50, 13.2%)	11.6 ± 11.6	10.2 ± 8.7	62.6 ± 30.8	49.9 ± 19.5	7.9 (4.1, 13.0)	7.5 (3.0, 12.0)
Chile (*n* = 1; *n* = 32, 69.6%)	SP	3.8 ± 8.2	SP	34.8 ± 17.1	SP	1.0 (0.0,2.0)
Costa Rica (*n* = 6; *n* = 8, 72.7%)	4.0 ± 0.0	18.6 ± 12.9	60.0 ± 0.0	45.0 ± 15.0	6.5 (2.1, 11.3)	8.0 (3.8, 25.0)**
Croatia (*n* = 3; *n* = 1, 33.3%)	SCS	SP	SCS	SP	SCS	SP
Czech Republic (*n* = 6; *n* = 11, 84.6%)	4.2 ± 4.6	6.0 ± 7.5	22.0 ± 22.6	40.0 ± 27.1	0.6 (0.3, 6.7)	2.0 (1.0, 2.0)
Denmark (*n* = 8; *n* = 3, 3.1%)	6.4 ± 4.0	SCS	111.0 ± 77.7	SCS	9.0 (4.4, 29.0)	SCS
England (*n* = 57; *n* = 27, 14.7%)	7.6 ± 5.0	7.8 ± 5.3	56.4 ± 50.1	52.6 ± 25.1	6.0 (4.0, 8.0)	5.0 (3.0, 8.0)*
Finland (*n* = 11; *n* = 7, 46.7%)	18.0 ± 18.4	3.6 ± 2.8	82.5 ± 21.2	60.0 ± 0.0	12.5 (8.3, 42.5)	2.0 (1.0, 6.0)**
France (*n* = 16; *n* = 22, 15.3%)	9.8 ± 5.4	12.2 ± 7.9	55.4 ± 16.3	56.8 ± 15.6	8.0 (6.4, 10.0)	10.0 (5.0, 15.0)**
Germany (*n* = 34; *n* = 9, 5.8%)	20.6 ± 30.7	12.3 ± 9.0	54.5 ± 41.0	49.3 ± 11.3	12.0 (5.5, 19.8)	8.5 (4.3, 14.0)**
Greece (*n* = 4; *n* = 6, 54.6%)	5.0 ± 4.1	5.6 ± 5.3	13.8 ± 12.5	22.5 ± 18.5	1.5 (0.3, 2.3)	1.0 (0.3, 2.5)
Hungary (*n* = 20; *n* = 6, 26.1%)	8.3 ± 6.1	9.0 ± 5.2	36.3 ± 13.1	51.7 ± 34.2	4.3 (1.4, 7.9)	6.5 (4.5, 8.8)*
Ireland (*n* = 7; *n* = 40, 87.0%)	12.6 ± 3.0	8.0 ± 4.6	58.5 ± 3.4	47.9 ± 14.4	13.0 (10.0, 14.0)	6.0 (4.0,9.0)**
Israel (*n* = 6; *n* = 2, 11.8%)	1.8 ± 0.8	SCS	54.0 ± 25.1	SCS	1.0 (1.0, 2.3)	SCS
Italy (*n* = 70; *n* = 9, 9.0%)	6.9 ± 7.4	6.3 ± 4.6	48.5 ± 26.1	40.0 ± 15.1	3.2 (1.5, 7.5)	3.0 (2.0, 7.0)
Japan (*n* = 9; *n* = 47, 6.9%)	5.2 ± 7.3	5.3 ± 11.1	38.6 ± 16.8	28.8 ± 18.4	1.7 (0.8, 7.3)	1.0 (0.0, 2.3)*
Lithuania (*n* = 9; *n* = 1, 0.5%)	6.3 ± 5.5	SP	49.2 ± 50.2	SP	2.5 (0.7, 12.1)	SP
Luxembourg (*n* = 0; *n* = 1, 25.0%)	–	SP	–	SP	–	SP
Malta (*n* = 1; *n* = 1; 100.0%)	SP	SP	SP	SP	SP	SP
Netherlands (& Aruba) (*n* = 29; *n* = 91, 100.0%)	5.6 ± 3.2	2.7 ± 2.0	69.2 ± 36.1	78.6 ± 22.9	5.1 (3.0, 8.3)	3.0 (3.0, 3.0)**
New Zealand (*n* = 27; *n* = 13, 38.2%)	5.8 ± 2.4	6.9 ± 6.3	72.3 ± 29.9	56.3 ± 27.2	6.0 (4.3, 9.0)	5.5 (2.3, 7.8)*
Northern Ireland (*n* = 10; *n* = 2, 22.2%)	8.1 ± 2.3	SCS	43.0 ± 8.6	SCS	5.0 (3.0, 10.0)	SCS
Norway (*n* = 0; *n* = 13, 54.2%)	–	19.4 ± 18.8	–	53.9 ± 16.7	–	8.0 (4.5, 26.0)
Panama (n = 1; n = 2, 100.0%)	–	SCS	–	SCS	–	SCS
Poland (*n* = 21; *n* = 26, 43.3%)	5.0 ± 4.1	4.9 ± 2.6	36.0 ± 11.3	37.9 ± 13.1	2.3 (1.9, 3.0)	3.0 (2.0, 4.0)*
Portugal (*n* = 21; *n* = 5, 17.9%)	13.7 ± 11.5	6.5 ± 3.4	56.1 ± 21.2	45.0 ± 17.3	8.0 (3.0, 25.0)	4.0 (2.3, 7.3)**
Qatar (*n* = 1; *n* = 1, 100.0%)	SP	SP	SP	SP	SP	SP
Romania (*n* = 2; *n* = 2, 8.7%)	SCS	SCS	SCS	SCS	SCS	SCS
Saudi Arabia (*n* = 0; *n* = 2; 50.0%)	–	SCS	–	SCS	–	SCS
Scotland (*n* = 24; *n* = 9; 52.9%)	6.3 ± 3.3	5.3 ± 2.9	42.0 ± 17.3	26.4 ± 13.1	3.8 (3.0, 5.6)	2.5 (0.5, 3.8)*
Singapore (*n* = 7; *n* = 7, 77.8%)	4.4 ± 4.0	4.0 ± 4.6	38.6 ± 24.3	19.0 ± 10.2	5.0 (0.5, 6.0)	1.0 (0.5, 1.5)**
Slovak Republic (*n* = 1; *n* = 1, 12.5%)	SP	SP	SP	SP	SP	SP
Slovenia (*n* = 2; *n* = 5, 50.0%)	SCS	11.0 ± 14.1	SCS	63.0 ± 26.8	SCS	5.0 (3.0, 31.0)
South Korea (*n* = 12; *n* = 32, 66.7%)	3.0 ± 3.0	4.4 ± 6.3	23.3 ± 6.2	32.5 ± 19.3	0.8 (0.5, 1.0)	1.0 (1.0, 2.0)
Spain (*n* = 47; *n* = 62, 67.4%)	9.5 ± 7.2	7.0 ± 6.9	51.2 ± 15.3	51.2 ± 14.2	6.0 (4.0, 9.3)	3.0 (2.0, 8.0)**
Sweden (*n* = 1; *n* = 30, 38.5%)	–	10.9 ± 8.9	–	54.7 ± 21.8	–	6.0 (3.3, 21.8)
Switzerland (*n* = 4; *n* = 1, 1.4%)	12.0 ± 0.0	SP	45.0 ± 0.0	SP	9.0 (9.0, 9.0)	SP
Taiwan (*n* = 23; *n* = 28, 53.9%)	7.6 ± 12.0	8.1 ± 12.4	22.9 ± 16.3	22.9 ± 11.7	1.0 (0.5, 1.6)	1.0 (0.0, 2.3)
United Arab Emirates (*n* = 0; *n* = 5, 71.4%)	–	17.3 ± 16.7	–	26.7 ± 15.3	–	6.0 (1.0, 6.0)
Uruguay (*n* = 5; *n* = 5, 21.0%)	3.7 ± 0.6	8.4 ± 9.4	44.0 ± 31.3	42.0 ± 19.6	4.0 (0.5, 4.0)	3.0 (1.0, 16.0)
United States of America (*n* = 65; *n* = 63, 2.3%)	14.6 ± 12.2	13.9 ± 11.6	42.1 ± 29.4	27.9 ± 17.9	6.0 (3.0, 12.0)	4.0 (2.0, 6.0)*
Wales (*n* = 16; *n* = 1, 8.3%)	7.6 ± 4.3	SP	53.6 ± 24.1	SP	6.0 (3.5, 9.0)	SP
**Upper-middle income (66.6%)**	**6.9 ± 7.8**	**6.4 ± 9.0**	**38.5 ± 25.5**	**32.9 ± 18.5**	**2.5 (1.0, 6.0)**	**2.0 (1.0, 4.0)**
Argentina (*n* = 3; *n* = 17, 70.8%)	SCS	6.0 ± 10.5	SCS	35.6 ± 18.4	SCS	0.5 (0.0, 7.3)
Azerbaijan (*n* = 0; *n* = 1, 33.3%)	–	SP	–	SP	–	SP
Brazil (*n* = 30; *n* = 49, 83.1%)	6.0 ± 10.5	7.0 ± 10.0	45.1 ± 29.6	39.5 ± 21.7	1.3 (0.3, 5.4)	2.0 (0.0, 3.0)
China (*n* = 83; *n* = 226, 61.4%)	4.2 ± 5.4	5.1 ± 7.2	31.7 ± 21.1	28.8 ± 14.1	1.0 (0.7, 3.0)	1.0 (1.0, 3.0)
Colombia (*n* = 48; *n* = 46, 100.0%)	6.0 ± 6.7	8.0 ± 11.8	49.1 ± 31.3	41.2 ± 19.6	3.0 (1.5, 6.0)	2.0 (1.0, 4.0)*
Cuba (n = 8; n = 3, 14.3%)	12.0 ± 10.2	SCS	37.5 ± 17.3	SCS	3.3 (3.0, 10.5)	SCS
Dominican Republic (n = 1; *n* = 2, 66.7.0%)	SP	SCS	SP	SCS	SP	SCS
Georgia (*n* = 13; *n* = 17, 94.4%)	8.4 ± 6.8	14.5 ± 11.5	20.0 ± 8.4	42.7 ± 16.5	1.7 (0.7, 5.8)	8.0 (1.0, 20.0)**
Guatemala (*n* = 2; *n* = 2, 50.0%)	SCS	SCS	SCS	SCS	SCS	SCS
Indonesia (*n* = 10; *n* = 13, 76.5%)	1.9 ± 1.8	2.0 ± 1.3	24.1 ± 18.0	29.1 ± 19.2	0.5 (0.3, 1.9)	1.0 (0.0, 1.0)*
Iran (*n* = 14; *n* = 5, 13.2%)	6.0 ± 5.5	12.0 ± 16.1	40.4 ± 20.9	63.8 ± 39.4	2.0 (1.4, 4.9)	3.5 (3.0, 28.0)**
Jamaica (*n* = 0; *n* = 1, 50.0%)	–	SP	–	SP	–	SP
Kazakhstan (*n* = 0; *n* = 10, 100.0%)	–	2.7 ± 3.4	–	31.7 ± 14.7	–	1.0 (0.0, 3.0)
Malaysia (*n* = 4; *n* = 6, 66.7%)	5.3 ± 3.1	5.0 ± 2.2	35.0 ± 8.7	37.0 ± 15.7	4.0 (1.0, 4.0)	3.0 (1.5, 5.5)**
Mexico (*n* = 9; *n* = 65, 100.0%)	8.1 ± 8.1	8.9 ± 10.3	34.4 ± 20.5	35.6 ± 22.1	4.0 (1.1, 5.5)	2.0 (1.0, 8.0)**
Moldova (*n* = 1; *n* = 1, 100.0%)	SP	SP	SP	SP	SP	SP
Montenegro (*n* = 0; *n* = 1, 100.0%)	–	SP	–	SP	–	SP
Paraguay (*n* = 3; *n* = 1, 50.0%)	SCS	SP	SCS	SP	SCS	SP
Peru (*n* = 7; *n* = 12, 100.0%)	12.8 ± 6.2	11.6 ± 18.3	75.4 ± 34.9	27.2 ± 15.8	14.5 (7.8, 27.0)	3.0 (1.0, 4.5)**
Serbia (*n* = 2; *n* = 5, 100.0%)	SCS	6.5 ± 7.8	SCS	27.5 ± 5.0	SCS	2.0 (1.0, 7.5)
South Africa (*n* = 14; *n* = 2, 8.7%)	13.0 ± 12.0	SP	30.5 ± 16.9	SP	4.8 (1.9, 6.8)	SP
Suriname (*n* = 0; *n* = 1; 100.0%)	–	SP	–	SP	–	SP
Thailand (*n* = 0; *n* = 12; 63.2%)	–	–	–	–	–	–
Turkey (*n* = 9; *n* = 10, 76.9%)	16.3 ± 12.9	11.8 ± 14.1	33.8 ± 20.3	37.0 ± 21.7	6.5 (0.8, 28.1)	1.0 (0.5, 27.0)**
**Lower-middle income (86.3%)**	**4.9 ± 4.2**	**4.9 ± 5.0**	**29.4 ± 19.5**	**30.9 ± 19.4**	**1.7 (0.7, 3.3)**	**2.0 (1.0, 4.0)**
Bangladesh (*n* = 1; *n* = 3, 75.0%)	5.0 ± 0.0	SCS	20.0 ± 0.0	SCS	1.7 (1.7, 1.7)	SCS
Cameroon (*n* = 0; *n* = 1, 14.3%)	–	SP	–	SP	–	SP
Cote d’Ivoire (*n* = 0; *n* = 5, 50.0%)	–	9.0 ± 2.0	–	46.3 ± 18.9	–	7.5 (3.5, 10.0)
Honduras (*n* = 1; *n* = 2, 66.7%)	SP	SCS	SP	SCS	SP	SCS
India (*n* = 18; *n* = 80, 100.0%)	3.9 ± 3.0	5.1 ± 6.1	24.3 ± 11.3	25.0 ± 14.8	1.0 (0.5, 2.5)	1.0 (1.0, 3.0)
Jordan (*n* = 0; *n* = 1, 100.0%)	–	SP	–	SP	–	SP
Kenya (*n* = 1; *n* = 4, 100.0%)	SP	2.8 ± 2.8	SP	31.7 ± 12.5	SP	2.0 (0.0, 2.0)*
Mauritania (*n* = 0; *n* = 1; 33.3%)	–	SP	–	SP	–	SP
Nigeria (*n* = 1; *n* = 3, 100.0%)	SP	SCS	SP	SCS	SP	SCS
Pakistan (*n* = 2; *n* = 8, 100.0%)	SCS	6.3 ± 4.8	SCS	42.5 ± 19.3	SCS	4.0 (2.0, 4.8)
Philippines (*n* = 10, 0.9%; *n* = 18, 1.2%)	5.4 ± 3.7	2.7 ± 2.1	29.0 ± 13.7	24.6 ± 14.0	2.1 (0.9, 3.3)	1.0 (0.0, 2.0)
Senegal (*n* = 0; *n* = 8; 100.0%)	–	6.4 ± 5.4	–	55.6 ± 29.9	–	3.5 (2.3, 12.5)
Sri Lanka (*n* = 2; *n* = 13, 100.0%)	SCS	5.5 ± 3.3	SCS	33.8 ± 27.4	SCS	2.5 (0.3, 6.8)
Tanzania (*n* = 0; *n* = 2, 100.0%)	–	SCS	–	SCS	–	SCS
Tunisia (*n* = 1; *n* = 4, 57.1%)	SP	5.5 ± 4.7	SP	32.5 ± 14.4	SP	3.0 (1.0, 8.0)*
Vietnam (*n* = 0; *n* = 3; 100.0%)	–	SCS	–	SCS	–	SCS
Zimbabwe (*n* = 0; *n* = 2, 100.0%)	–	SCS	–	SCS	–	SCS
**Low income (66.7%)**	**–**	**NA**	**–**	**NA**	**–**	**NA**
Malawi (*n* = 0; *n* = 15, 65.2%)	–	4.2 ± 3.1	–	16.6 ± 8.1	–	1.0 (1.0, 1.75)
Sudan (*n* = 0; *n* = 1, 100.0%)	–	SP	–	SP	–	SP
**Not classified (NA)**	**–**	**4.1 ± 3.8**	**–**	**33.9 ± 16.0**	**–**	**2.0 (1.0, 3.5)**
Venezuela (*n* = 8; *n* = 5, 100.0%)	10.4 ± 3.4	5.7 ± 4.3	46.9 ± 12.5	48.8 ± 14.4	7.5 (5.3, 11.5)	3.5 (2.0, 8.0)**
Unknown (NA; *n* = 18, NA)	NA	3.3 ± 3.2	NA	32.3 ± 16.7	NA	1.0 (1.0, 2.5)
**Global (*N* = 1,505)** ^c^	**8.5 ± 10.8**	**6.9 ± 8.4**	**47.0 ± 29.7**	**41.1 ± 23.3**	**4.5 (1.6, 8.0)**	3.0 (1.0, 7.0)**

–No programs, or the education dose items were not complete among surveys received.

SP: data Suppressed to protect program Privacy (i.e., only 1 program in country).

SCS: data suppressed due to Small Cell Sizes rendering estimates unreliable (≤3 programs responding).

^a^Per patient, per completed prescribed supervised program.

^b^Mann–Whitney *U* tests were used to compare total education dose between 2016 and 2025 Audit where data available for both decades. Differences were considered statistically significant at *p* < 0.01* and *p* < 0.001** to consider high number of comparisons.

^c^Generalized Linear Mixed Model in 2025 data, with post-hoc pairwise tests revealing significantly higher supervised exercise doses offered in high income compared to any middle-income countries (as detailed in Results section; unclassified surveys not considered).

Acronyms: NA, not available or applicable.

**Table 2:** Risk factors assessed in phase II cardiac rehabilitation initial assessment by World Bank country income classification [25] in 2025 and audit decade globally.

Risk Factor	World Bank Country Income Classification (n, program response rate in income class)				Audit Decade^a^	
	High *n* = 796 (13.3%)	Upper-middle *n* = 510 (66.6%)	Lower-middle *n* = 159 (86.3%)	Low *n* = 17 (66.7%)	Global 2016 *N* = 1,082	Global 2025^b^ *N* = 1,505
Blood pressure	686 (99.4)	456 (99.6)	126 (100.0)	15 (100.0)	928 (99.1)	1,300 (99.5)
Physical activity	674 (98.3)	449 (98.5)	121 (97.6)	15 (100.0)	879 (94.3)	1,276 (98.3)**
Tobacco use	676 (99.0)	448 (98.0)	114 (95.8)	11 (91.7)	919 (98.0)	1,265 (98.2)
Poor diet	633 (93.0)	427 (94.1)	111 (94.1)	11 (91.7)	840 (90.1)	1,199 (93.5)*
Harmful use of alcohol and/or drugs	626 (93.0)	423 (93.8)	107 (90.7)	10 (66.7)	867 (92.5)	1,181 (93.0)
HbA_1_c and/or blood glucose	595 (88.9)	424 (93.4)	114 (95.8)	6 (60.0)	676 (72.2)	1,156 (91.1)**
Lipids	599 (89.7)	433 (94.7)	107 (88.4)	1 (11.1)	853 (91.03)	1,156 (90.9)
Depression	608 (89.3)	395 (87.8)	87 (76.3)	10 (66.7)	797 (85.8)	1,117 (87.5)
Time spent being sedentary	526 (79.5)	391 (87.3)	93 (79.5)	10 (66.7)	708 (76.4)	1,034 (82.3)**
Body composition^c^	446 (67.6)	353 (79.1)	80 (69.6)	4 (36.4)	900 (95.8)	896 (71.8)
Sleep apnea	458 (69.5)	337 (75.9)	67 (60.4)	5 (45.5)	437 (47.4)	878 (70.8)**
Other psychosocial (e.g., support)^d^	572 (85.0)	323 (73.2)	79 (68.1)	10 (66.7)	–	998 (79.1)
Erectile dysfunction^d^	299 (47.5)	160 (37.2)	39 (36.4)	2 (20.0)	–	504 (42.3)
Other	146 (35.0)	104 (28.4)	10 (14.9)	1 (14.3)	213 (39.3%)	263 (30.4)
**Total Number of Risk Factors Assessed (mean ± SD/10)** ^a^^,^^d^	**7.6 ± 3.4**	**8.2 ± 3.1**	**6.6 ± 3.8**	**5.5 ± 3.0**	**7.3 ± 3.2**	7.9 ± 3.4*

Note 1: *n*, valid % shown, by descending frequency in 2025. Due to missing data, percentages are computed with the number of valid responses from responding programs as the denominator.

Note 2: significant differences by country income class are shown in Fig 3.

–Not available.

^a^Chi-squared tests were used to compare differences between 2016 and 2025 Audit. Differences were considered statistically significant at *p* < 0.01* and *p* < 0.001** given number of comparisons.

^b^Eighteen surveys with unknown country and 5 surveys in countries that are not income level classified (i.e., Venezuela) included in Global column only.

^c^There were separate response options for body mass index and waist circumference in the 2016 survey, which are shown conglomerated. Decade change not assessed due to measurement change.

^d^Total does not include other psychosocial factors or erectile dysfunction as not assessed in initial Audit.

Abbreviation: HbA1c, hemoglobin A1c.

Acronyms: SD, standard deviation; CI, confidence interval.

Survey-responding CR staff were most commonly: medical directors (*n* = 576, 38.3%), followed by allied healthcare professionals (*n* = 396, 26.3%), or nurses (*n* = 180, 12.0%). Other respondent team members were: cardiac technologists (*n* = 3), researchers (*n* = 3), and physicians-in-training (*n* = 2).

Overall, 1,327 (89.4%) programs were situated in urban areas. Moreover, 1,135 (78.7%) were located within hospitals, of which 985 (87.1%) had an inpatient cardiology unit. CR program location differed significantly by country income class, with low-income countries more often having programs in hospital/tertiary care settings (93.3%, 95% confidence interval [CI; 70.2,98.8%]; *χ*² = 60.7, *p* < 0.001). The proportion of programs in hospitals with an inpatient cardiology service did not differ significantly across income classes (*χ*² = 0.90, *p* = 0.92). Note that in the initial 2016 Audit, 775 (72.8%) programs were situated in urban areas, and 851 (80.6%) were located within hospitals [17].

### Program elements delivered

**Fig 2:**
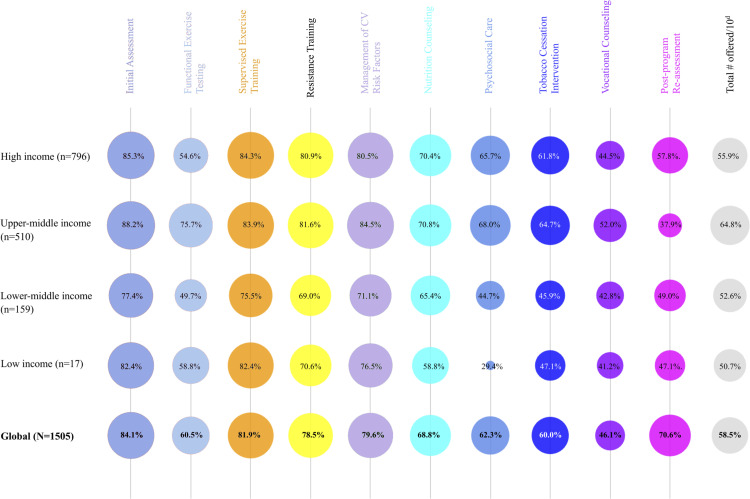
Core components^a^ offered in 2025 by phase II cardiac rehabilitation programs^b^ by World Bank country income classification [25] and globally^c^. Acronym: CV, cardiovascular. Note: The size of each colored ball reflects the proportion of programs in that income class delivering that component. ^a^Some response options from the survey were grouped to fulsomely reflect component offerings. Initial assessment comprised responses of: assessment of cardiac history and risk, individual consult with a physician or nurse, and/or assessment of comorbidities/ issues that could impact exercise. Functional exercise testing included an exercise stress test, other type of functional capacity test assessment of strength, and/or exercise prescription. Management of CV risk factors also considered prescription and/or titration of secondary prevention medications. Psychosocial care comprised psychological assessment, sexual health assessment, stress management/relaxation techniques, and/or end-of-life counseling. Post-program re-assessment also considered communication of patient assessment results to primary care and/or follow-up after outpatient program. Initial assessment was part of program inclusion criteria, but detailed aspects of assessment assessed and shown in Figure. ^b^Programs also reported where they referred patients elsewhere to receive each component (shown in Table 3); only affirmative ‘yes’ responses shown in Figure. ^c^18 surveys with unknown country and 5 surveys in countries that are not income level classified (i.e., Venezuela) included in Global row. ^d^ Kruskal–Wallis test revealed significant differences in total number of components delivered (*p* < 0.001) between: (a) programs in upper-middle income and high income (*p* = 0.01), lower-middle income (*p* < 0.001), and low income (*p* < 0.001) countries; and (b) high income and low income (*p* = 0.01) countries.

Fig 2 displays the core components delivered by CR programs globally, with Table 3 presenting this for key components by country; note no “other” elements were commonly reported. As also shown in Table 3, programs did on average refer patients elsewhere for almost 2 elements, most commonly for end-of-life counseling, sexual health, alternative forms of exercise, tobacco cessation, prescriptions and titration of medications, women-focused classes, and return-to-work supports.

**Table 3:** Phase II cardiac rehabilitation components and other elements delivered by programs by audit decade globally.

CR Elements	Global 2025 *N* = 1,505		Global 2016 *N* = 1,082
	Patient Referred Elsewhere	Program Offered	Program Offered
Initial assessment^a^^,^^b^	9 (0.7)	1,283 (99.1)	939 (98.8)
Individual consultation with a physician or nurse	53 (4.1)	1,164 (90.7)	897 (94.4)
Exercise stress test	124 (9.8)	922 (73.1)	656 (70.0)
Other functional capacity test	70 (5.6)	937 (74.7)	734 (78.9)
Assessment of strength (e.g., handgrip)	47 (3.8)	792 (63.6)	138 (57.3)
Assessment for comorbidities/issues that could impact exercise	45 (3.5)	1,156 (90.7)	877 (93.1)
Exercise prescription	9 (0.7)	1,251 (97.2)	918 (97.0)
Supervised exercise training^a^^,^^b^	14 (1.1)	1,249 (96.7)	898 (94.3)
Resistance training	16 (1.3)	1,197 (93.7)	858 (90.8)
Patient education^a^	9 (0.7)	1,257 (97.9)	895 (96.9)
Management of cardiovascular risk factors^a^	37 (2.9)	1,215 (95.1)	928 (98.2)
Prescription and/or titration of secondary prevention medications	184 (14.6)	929 (73.9)	751 (79.3)
Nutrition counseling^a^	125 (9.8)	1,047 (82.4)	880 (92.7)
Psychological assessment (e.g., depression)^a^	173 (13.7)	951 (75.1)	816 (86.3)
Tobacco cessation intervention and/or counseling^a^	212 (16.8)	915 (72.6)	692 (73.3)
Sexual health assessment	247 (20.3)	426 (35.0)	NA
Vocational counseling/ support for return-to-work^a^	188 (12.5)	702 (56.7)	614 (65.7)
Stress management/ relaxation techniques	115 (9.1)	930 (73.9)	771 (81.7)
End-of-life counseling	280 (23.2)	274 (22.7)	NA
Alternative forms of exercise (e.g., yoga, dance, tai chi)	224 (18.3)	441 (36.1)	355 (38.0)
Women-focused classes	164 (13.5)	187 (15.4)	110 (11.8)
Inclusion of family/ informal caregivers	91 (7.3)	791 (63.7)	NA
End of program re-assessment^a^	44 (3.5)	1,076 (86.3)	858 (91.4)
Communication of patient assessment results with primary care provider^a^	49 (3.9)	993 (79.9)	788 (84.0)
Follow-up after outpatient program	90 (7.3)	830 (67.5)	662 (70.4)
Maintenance program^c^ (phase III/IV)	136 (11.0)	709 (57.4)	NA
**Total core**^a^^,^^b^ **(median [p25, p75]/10)**	**0.0 (0.0, 1.0)**	**8.0 (6.0, 10.0)**	**9.0 (7.0, 10.0)**
**Total core**^a^^,^^b^ **(mean ± SD/10)**	**0.6 ± 1.2**	**7.1 ± 3.3**	**7.5 ± 3.2**
**Total elements**^c^ **(median [P25,P75]/22)**	**0.0 (0.0, 0.0)**	**19.0 (15.0, 22.0)**	**17.0 (14.0, 19.0)**
**Total elements**^c^ **(mean ± SD/22)**	**1.3 ± 2.8**	**17.7 ± 8.9**	**15.1 ± 6.2**

Note: *n*, % shown. Due to missing data, percentages are computed with the number of valid responses from responding programs as the denominator.

^a^Core components included are: initial assessment, management of risk factors, supervised exercise training, patient education, nutrition counseling, psychological counseling, smoking cessation intervention, vocational counseling, end of program reassessment, and communication of assessment results [10].

^b^Inclusion criteria for programs was to offer at least initial assessment and structured aerobic exercise (not necessarily supervised), and ≥1 other core component to control risk factors.

^c^The four components not assessed in 2016 survey were not included in total, nor was the “other” response option.

Acronyms: CR, cardiac rehabilitation; NA, not assessed; SD, standard deviation.

Not considering the above, the median number of core components offered was 8.0 (6.0–10.0) of 10, and of elements offered per program was 19.0 (p25, p75 = 15.0, 22.0) of 22 (Table 3). Not considering initial assessment, which was a program inclusion criterion, the CR components patient education, exercise prescription, supervised exercise training and management of CV risk factors were the most-frequently implemented across programs currently globally, with sexual health assessment, end-of-life counseling, and women-focused classes least. Note that globally, over one-third of programs offered alternative forms of exercise, and just under two-thirds included informal caregivers. At program end, globally, four-fifths of programs communicated a summary of patient status to the referring clinician, and two-thirds of programs followed up with patients post-program. Finally, well over half of programs offered a phase III maintenance program (Table 3).

Main component delivery frequency by income class is illustrated in Fig 2 and S1 Table. There was a significant difference in the total number of components offered by income class (Kruskal–Wallis test; *H* = 19.369, *p* < 0.001), with post-hoc comparisons showing that upper-middle income countries offered more components overall than high income (*p* = 0.01), lower-middle income (*p* < 0.001), and low-income countries (*p* < 0.001). Moreover, programs in high-income countries offered more components than programs in low-income countries (*p* = 0.01).

Comparison of findings from this 2025 Audit with the initial 2016 Global CR Audit revealed much consistency in the frequency of elements offered by programs over the past decade (Table 3; additional details regarding these offerings by country during the initial Audit are available in the previously published work [17]). Note again, however, that there was a response option of “patient referred elsewhere” to receive each element in the Audit Update survey; considering that change, inferential tests were not performed. However, several CR components, namely supervised exercise training, resistance training, and women-focused classes, appeared to be delivered more frequently in the current era.

**Fig 3:**
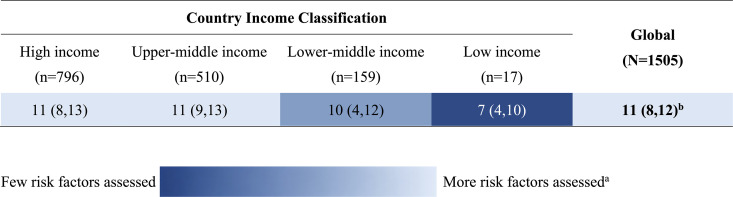
Heat map of total number of risk factors assessed^a^ in 2025 by World Bank country income classification [25] and globally. Note^:^ median (p25, p75) shown. ^a^Of 14 assessed. ^b^Difference in median number of risk factors assessed were tested by country income class using linear mixed models accounting for clustering of programs within countries, *p* < 0.001; Bonferroni-adjusted post-hoc comparisons: High and upper-middle-income countries assessed significantly more risk factors than lower-middle countries (all *p* < 0.01; differences with low-income not tested given data from only one country available).

Risk factors assessed during the initial assessment are depicted in Table 2 (median total in Fig 3). Globally, in descending frequency, blood pressure, physical activity, tobacco use, diet, and use of substances were among the most-commonly assessed, with erectile dysfunction the least commonly assessed in the current era (shown by country in S2 Table). Other risk factors listed by responding programs included: medication non-adherence (*n* = 12), family history (*n* = 12), socioeconomic status (*n* = 8), hormonal history and related factors among women (*n* = 6), sleep quality (*n* = 5), novel risk factors under study (e.g., Lp(a); *n* = 4), and occupational work stress.

Table 2 also shows the frequency of assessment of key risk factors by country income classification, with total number assessed shown in Fig 3. GLMM accounting for clustering of programs within countries showed that the number assessed per program varied significantly by country income class (*F*[4,1,477]=10.16, *p* < 0.001). Post-hoc comparisons showed that programs in lower-middle income countries assessed significantly fewer risk factors than those in both upper-middle-income (mean difference = 2.2, 95% CI [9.7,10.4]; *p* < 0.001) and high-income countries (mean difference = 1.6, 95% CI [9.2,9.8]; *p* < 0.001; differences with low-income not tested given data from only one country available). An examination of the frequencies suggests this may have been driven by less assessment of lipids, harmful use of substances, sleep apnea, and psychosocial risk factors (see also S2 Table).

Table 2 presents the risk factors assessed during Phase II CR initial assessments by Audit decade. Compared with the 2016 audit, programs in 2025 assessed fewer risk factors overall (mean ± standard deviation: 7.9 ± 3.4 versus 7.3 ± 3.2/10; *p* = 0.005; Table 2). At the individual risk factor level, programs in 2025 were significantly more likely to assess physical activity, diet, HbA_1_c/blood glucose, sedentariness, and sleep apnea; no other differences were observed.

### Patient education characteristics

As shown in Table 3 and Fig 1, virtually all existing programs in all countries with CR worldwide deliver patient education (exceptions are programs in Bahrain, Honduras, Jordan, and Zimbabwe with unknown data). In supervised models, the median number of sessions offered per patient was 4 (p25, p75 = 2.0, 8.0), and each session lasted a median of 30 min (p25, p75 = 20.0, 60.0; means shown in Table 1). Accordingly, the overall median education dose was 3.0 hours, which by country ranged from a maximum mean of 8.0 hours per program in Norway to a minimum of 1.0 hour per program in several countries, including Singapore, the Slovak Republic, Vietnam, and Jamaica (Table 1 and Fig 1).

### Patient education in home-based and/or hybrid models

In the third of programs globally where alternative/ hybrid program models were offered, with regard to service reimbursement (versus patients paying out-of-pocket), synchronous group education sessions via video were covered in 70 (18.9%) programs in 21 countries (Table 4 reports both by country income classification). In over two-thirds (67.9%) of home-based or hybrid program models, patients were provided with supporting online educational materials (Table 4 reports also presents this by country income classification).

**Table 4:** Global availability of alternative/hybrid cardiac rehabilitation models in programs, including synchronous video education coverage and supporting online educational materials, by World Bank country income classification [25] and globally.

Income Classification	Availability of Alternative/Hybrid CR Models	Coverage/Reimbursement of Synchronous Video Education^a^	Provision of Online Educational Materials^b^
High income	173 (26.3)	35 (20.2)	124 (71.7)
Upper-middle income	154 (30.2)	31 (20.1)	104 (67.5)
Lower-middle income	40 (35.1)	3 (7.5)	24 (60.0)
Low income	2 (15.4)	0 (0.0)	0 (0.0)
**Global**	**371 (29.5)**	**70 (18.9)**	**252 (67.9)**

Note: *n* (valid %) shown.

^a^Of programs who reported hybrid model reimbursement.

^b^Of programs offering alternative/ hybrid models.

Acronyms: CR, cardiac rehabilitation.

Table 1 presents the overall dose of the education component in supervised CR, stratified by income class. The GLMM model assessing total education dose differences by income class was statistically significant (*F*(4,1,184) = 10.066, *p* < 0.001). After adjustment for multiple comparisons, post-hoc pairwise comparisons demonstrated that programs in high-income countries delivered significantly higher doses than programs in both upper-middle (mean difference = 1.7 hours, 95% CI [3.6,5.1]; *p* = 0.002) and lower-middle income countries (mean difference = 3.0 hours, 95% CI [2.2,3.9]; *p* < 0.001).

Table 1 also presents the overall dose of the education component in supervised CR stratified by Audit decade. The global median total education dose per program was lower in 2025 (3.0 hours [p25, p75 = 1.0, 7.0]) than 2016 (4.5 hours [p25, p75 = 1.6, 8.0]). While not tested inferentially given the large number of countries, total education dose appears to have particularly decreased over the decade in Austria, Finland, Germany, Scotland, Peru, South Africa, Nigeria, and Venezuela. Conversely, increases in total education dose were noted in Belgium, France, Turkey, and South Korea.

### Program resources and other characteristics

Table 5 presents program resources (dedicated or shared with another service) globally by World Bank country income classification [25] and Audit decade. Across programs, the most-commonly available resources dedicated to CR currently were: bicycle ergometer (*n* = 862; 67.0%), resistance training equipment (*n* = 819; 64.6%), and gym space (*n* = 786; 61.9%). The resources most frequently shared with another service were: staff meeting rooms (*n* = 510; 41.2%), staff office space (*n* = 484; 39.1%), and gym space (*n* = 399; 31.4%). But overall, the most-commonly available program resources globally included gym space, resistance training equipment and individual assessment/counseling space (all >90%); least available were body composition analyzers and cardiopulmonary exercise testing equipment. Programs reported a median of 11 resources overall (p25, p75 = 8.0, 14.0). Other program resources listed by respondents included: equipment to deliver natural therapies (e.g., Traditional Chinese Medicine; *n* = 20), other types of exercise machines (e.g., rowing, elliptical; *n* = 7). outdoor exercise space (*n* = 6), cooking demonstration space or cafeteria space for nutrition education (*n* = 5), fitness class equipment (e.g., steps, *n* = 5), and swimming facilities (*n* = 4).

**Table 5:** Phase II cardiac rehabilitation program assets by World Bank country income classification [25] in 2025 and globally by audit decade.

Assets	World Bank Country Income Classification				Audit Decade^a^	
	High *n* = 796	Upper-middle *n* = 510	Lower-middle *n* = 159	Low *n* = 17	Global 2016 *N* = 1,082	Global 2025^b^ *N* = 1,505
Gym space	642 (95.3)	412 (91.2)	106 (90.6)	12 (85.7)	862 (93.6)	1,185 (93.4)
Resistance training equipment	621 (93.1)	416 (92.2)	111 (93.3)	14 (100.0)	790 (85.6)	1,178 (93.0)*
Individual assessment/Counseling room	598 (90.7)	417 (92.3)	108 (90.8)	12 (85.7)	855 (92.8)	1,151 (91.3)
Bicycle ergometer	639 (94.2)	382 (83.2)	106 (86.9)	9 (75.0)	821 (88.9)	1,150 (89.4)
Staff office space	593 (90.0)	369 (83.9)	92 (80.0)	9 (75.0)	803 (87.9)	1,075 (83.8)
Treadmill ergometer	536 (80.4)	396 (87.4)	114 (94.3)	8 (66.7)	773 (84.2)	1,068 (84.2)
Group education room	560 (85.0)	357 (81.3)	99 (83.9)	6 (60.0)	828 (89.9)	1,037 (83.6)*
Staff meeting room	544 (83.2)	358 (81.4)	94 (81.0)	9 (75.0)	751 (82.0)	1,019 (82.4)
Patient change room(s)	520 (79.3)	352 (79.3)	94 (80.3)	6 (60.0)	741 (80.4)	985 (79.2)
Electronic patient charts	516 (79.5)	372 (84.7)	61 (57.0)	2 (20.0)	559 (61.5)	959 (78.9)**
Administrative office	481 (73.1)	346 (78.1)	89 (76.7)	8 (66.7)	748 (81.5)	935 (75.2)**
Cardiac telemetry	340 (54.4)	305 (71.3)	64 (60.4)	0 (0.0)	513 (56.4)	715 (60.7)
Stress test (no O_2_)	292 (48.1)	342 (79.4)	69 (67.6)	4 (36.4)	541 (59.6)	717 (60.5)
Arm cyclo-ergometer	370 (59.0)	262 (60.9)	72 (66.7)	7 (70.0)	508 (56.4)	717 (60.5)
Stress test (with O_2_)	240 (39.8)	295 (70.9)	49 (47.6)	1 (10.0)	380 (42.6)	592 (51.9)*
Body composition analyzer	250 (40.5)	311 (70.8)	53 (48.2)	2 (20.0)	351 (38.6)	629 (52.9)**
Other	95 (27.9)	106 (31.3)	8 (12.9)	2 (20.0)	102 (25.9)	211 (27.9)
**Total number of assets/ 17 (mean±SD)** ^c^	**9.9 ± 4.8**	**11.4 ± 5.0** ^d^	**8.7 ± 5.4**	**6.5 ± 4.4**	**10.1 ± 5.0**	**10.2 ± 5.0**

Note: *n*, valid % shown, by descending frequency in 2025. Due to missing data, percentages are computed with the number of valid responses from responding programs as the denominator.

Acronym: SD, standard deviation.

^a^Chi-squared tests were used to compare differences between 2016 and 2025 Audit. Differences were considered statistically significant at *p* < 0.01* and *p* < 0.001** given number of comparisons

^b^Eighteen surveys with unknown country and 5 surveys in countries that are not income-level classified (i.e., Venezuela) included in Global column only.

^c^Generalized linear mixed model revealed statistically significant difference by income class, as fully described in text.

^d^Based on post-hoc pairwise comparisons with Bonferroni adjustment, total program assets were significantly greater among programs in upper-middle income programs than in programs in any other income class (all *p* < 0.001).

A GLMM was conducted to test for significant differences in total number of dedicated or shared resources by income class in the current era (Table 3). The model was statistically significant (*F*(4,1,477)=13.872, *p* < 0.001). Post-hoc pairwise comparisons indicated that programs in upper-middle income countries had significantly more resources than those in high (mean difference = 1.5, 95% CI [9.5,10.2]), lower-middle-income (mean difference = 2.6, 95% CI [8.0,9.5]), and low-income (mean difference = 4.8, 95% CI [4.2,8.9]; all *p* < 0.001). The resources that seemed to be driving these differences appeared to be related to availability of electronic patient charts, cardiac telemetry, stress testing equipment (with or without oxygen assessment), and body composition analyzers.

There were no significant differences by Audit decade overall (Table 3). However, with application of a conservative threshold of *p* < 0.01 to mitigate the number of comparisons, there were significant increases in program availability of the following resources: resistance training equipment, electronic patient charts, body composition analyzers, and stress testing with O_2_ in 2025 compared with 2016. In contrast, a significant decrease was observed in the availability of administrative office space and group education room.

Globally, 934 (76.8%) program staff reported assessing their care quality regularly, with such team performance evaluation highest in high-income (*n* = 502; 63.1%) and upper-middle income countries (336; 65.9%), but lower in lower-middle (*n* = 82; 51.6%) and low-income countries (*n* = 5; 29.4%). Moreover, 714 (61.7%) staff reported their programs were certified or their teams were interested in pursuing certification. Of these programs, 334 (42.0%) were in high-income, 297 (58.2%) in upper-middle, 72 (45.3%) in lower-middle, and 2 (11.8%) in low-income countries. In addition, 314 (27.1%) programs reported they would only seek certification if they had the human resources to do so (most common in high-income [*n* = 151; 19.0%] and upper-middle-income [*n* = 117; 22.9%] countries).

Globally, 704 (61.0%) program staff reported their programs were participating in a CR registry or their teams were interested in participation, and 245 (21.2%) programs reported they would participate if they had the resources to do so (most common in upper-middle- and high-income countries; 79.8% and 74.9%, respectively). Reasons program staff selected for not being part of a registry were: lack of staff with extra time for approvals and training (*n* = 222, 14.8%), lack of staff time for data entry and patient assessments (*n* = 215, 14.3%), insufficient capacity to follow-up with patients (*n* = 110, 7.3%), limited technological proficiency or literacy of patients (*n* = 93, 6.2%), and lack of institutional support (*n* = 68, 4.5%); “other” reasons included limited resources and equipment, regulatory barriers, financial disincentives, lack of awareness about the registry, and competing institutional priorities.

Finally, globally, 926 (75.8%) programs were at least somewhat familiar with ICCPR, with familiarity highest in upper- and lower-middle income countries (83.1% and 82.5%, respectively).

## Discussion

This 2025 Global CR Audit Update offers the only comprehensive assessment of CR program delivery worldwide—not only since the COVID-19 pandemic, but also since the initial Audit almost a decade ago - offering an exclusive global perspective on the current status of CR services and how they have evolved. With participation from even more programs in 2025 (*N* = 1,505, median of two-thirds of programs responding per country) across 90 countries (80% of all countries with CR), overall results of this Audit demonstrate the continued quality of CR care worldwide despite the disruptions and health resource constraints of the past decade [18]. The findings reveal comprehensive delivery of guideline-recommended core CR components globally (median 8/10, with patients referred elsewhere for some components), though variation persists favoring higher income countries [27]. Moreover, programs assessed on average 79% of risk factors evaluated. Finally, programs had an average of 10 resources (e.g., equipment, dedicated spaces) to enable rigorous delivery of these components. In all cases, however, higher-resourced settings were able to assess more risk factors in addition to delivering more components with these additional resources when compared to lower-resource programs.

Across programs worldwide, delivery of the fundamental CR elements remains robust, with a median of eight of 10 core components implemented. Patient education, exercise prescription and supervision, and management of cardiovascular risk factors were the most consistently offered components globally (in addition to initial assessment), reflecting strong adherence to guideline-based standards [4]. In contrast, components such as sexual health assessment, end-of-life counseling, and women-focused classes were less frequently available.

Programs in upper-middle-income countries reported offering a greater number of components overall than those in other income groups. This could reflect, first, that programs opened more recently in these contexts [16], such that delivery practices reflect more current evidence-informed practice standards. Second, Societies in these contexts have been developing local CR guidelines for the first time [15], likely positively impacting care quality. Third, patients in these contexts often have higher rates of multiple, uncontrolled risk factors [28], requiring comprehensive component delivery for secondary prevention. Finally, programs in these contexts are more often resourced by private funding sources as well as public ones, which may affect breadth capacity or requirements; further research is needed to understand thhe reasons. While programs in high-income countries also exceeded the comprehensiveness of those in low-income settings, programs across all contexts demonstrated substantial breadth of service delivery. Notably, even in lower-resource settings, programs are delivering the majority of recommended elements, which is impressive given the resource and system constraints often faced [29].

Regarding patient education, virtually all programs worldwide continue to provide this foundational element, affirming its recognized role in risk factor management and behavioral change [30,31]. However, the Audit revealed a significant reduction in education dose compared with 2016, particularly in high-income countries. While this may partly reflect transition to digital delivery [32] or better tailoring to individual patient risk factors, it also raises concerns about adequate time allocation for comprehensive education, particularly for patients with lower digital or other forms of literacy who may need the education most [33]. Consistent with this shift, the number of spaces dedicated to group education sessions also decreased, suggesting that digital or hybrid modalities are increasingly being used to deliver educational components remotely. Programs in high-income countries offered the greatest education dose overall, yet many middle- and lower-income countries are effectively leveraging group formats and hybrid delivery to extend reach. That over two-thirds of programs in home-based or hybrid models provide supporting materials—and that some synchronous education sessions are now reimbursed—is promising evidence of innovation toward scalable, equitable patient education.

Programs worldwide continue to assess a broad range of CV risk factors during initial evaluation, reflecting strong adherence to comprehensive prevention standards. Blood pressure, physical activity, tobacco use, nutrition, and harmful substance use were most consistently evaluated. The total number of risk factors assessed differed significantly by country income classification, with programs in high- and upper-middle-income countries assessing more factors than those in lower-middle–income settings. This disparity appears largely driven by less frequent evaluation of lipids, substance use, sleep apnea, and psychosocial risk factors in lower-resource contexts; some of these differences likely reflect health system organization, such as lipid panels often requiring out-of-pocket payment in these settings. Encouragingly, since the 2016 Audit, there have been notable increases in assessment of physical activity, nutrition, glycemic control, sedentariness, and sleep apnea, underscoring growing global attention to behavioral and metabolic risk. Similarly, the number of available program resources varied significantly by income class, with programs in upper-middle-income countries reporting the greatest number of dedicated and shared resources, including electronic patient charts, cardiac telemetry, and stress testing equipment. In contrast, programs in low-income settings operated with fewer resources, though the most essential facilities (e.g., gym space, resistance training equipment, and counseling rooms) were widely available across all contexts.

Taken together, these findings have important implications for practice, policy, and research. With regard to clinical practice, first, despite progress, gaps remain in the consistent delivery of core CR components, particularly in lower-middle and low-income countries. Clinically, CR teams should continue to embed comprehensive risk assessment and education strategies as standard, not optional, components of care. International collaborations could facilitate resource sharing, such as multilingual educational materials, standardized digital modules, and telehealth infrastructure adaptable to local capacity [34–37].

Patient education remains a critical element of CR, and programs should focus on ensuring that education is evidence-based and tailored to patient needs. The adoption of hybrid and home-based models offers opportunities to extend educational reach [36,38], but attention must be given to maintaining accessibility and quality, particularly in resource-constrained settings. Notably, only 18.9% of hybrid programs currently deliver education using video or virtual CR best practices, highlighting a gap in leveraging technology to optimize patient learning. Equitable access to dedicated education spaces and materials should be recognized as essential to ensuring program quality [39].

From a policy perspective, these disparities highlight the need for coordinated national and international strategies that prioritize equitable access to comprehensive, well-resourced CR programming. At the national and health-system levels, government health Ministries, hospital systems, and program administrators should consider mechanisms to support programs in under-resourced contexts, for example, through capacity-building initiatives, investment in program assets, and training for multidisciplinary teams. At the payer and commissioning level, insurers and health service funders should ensure adequate coverage for core CR components, including home-based education and digital delivery modalities. At the professional and knowledge translation level, guideline committees and international organizations should support quality assessment mechanisms, standards development, international collaboration, and knowledge sharing to reduce disparities in care delivery.

Finally, based on this updated Audit, some priorities for future research arise. Specifically with regard to education, research is needed to develop more comprehensive and standardized approaches to measuring patient education in CR, considering not only formal structured sessions, but also informal or embedded education delivered during routine clinical encounters throughout a program. This would better capture the full scope of educational exposure, as well as improve assessment comparability across programs and settings. Moreover, observed reductions in reported hours of formal patient education across programs warrant further investigation, including whether these changes reflect a shift toward digital or hybrid modes of education delivery over the past decade, a shift towards more informal delivery, as well as how this continues to involve in future. Importantly, the impact of education mode and level of formality on patient knowledge, behavior, and secondary prevention remains to be elucidated. Ideas related to assessment are expounded below.

Critical broader directions for future research include evaluating strategies to improve the delivery of core components in under-resourced settings and monitoring progress in equity-focused initiatives. Research should also explore innovative, scalable approaches to support consistent and high-quality CR care globally, ensuring that all patients, irrespective of country income classification, can benefit from evidence-based secondary prevention.

Caution is warranted in interpretation of these findings, particularly with respect to generalizability and potential sources of bias. Response rates to online surveys are often modest; while the country-level response in this Audit was high, program-level participation was low in some jurisdictions (although more representative than the initial Audit). Some countries did not respond, and several had only a small number of programs represented, limiting the ability to draw firm conclusions about those contexts. Particular caution is warranted in interpretation of results from low-income countries; while it is encouraging there is now CR in any of these countries since the first Audit [16,19], responses were received from only 16 programs in 2/6 low-income countries with CR. Complete identification of all programs was also challenging, particularly in countries without CR Societies or centralized program lists. Consequently, ascertainment bias is possible, as programs were likely more comprehensively identified in countries with established CR networks and longer-standing services. Moreover, since some national Societies distributed the survey through their email lists, the denominator of unique program invitations is uncertain, precluding precise consent rate calculation.

Program selection bias may also have occurred; whereby more established or higher-quality programs were more likely to participate despite survey confidentiality. Although efforts were made to prevent duplicate responses from the same programs (e.g., explicit instructions in invitation letters and data screening for anomalies), some duplication cannot be ruled out. Furthermore, while multilevel models accounted for clustering of programs within countries, marked variation in the number of participating programs per country may have introduced response bias. Countries with a greater number of programs (often higher-income settings) contributed disproportionately to global estimates, limiting generalizability to regions with fewer or resource-constrained programs.

Measurement-related limitations also apply. While delivery of any patient education by a program was assessed, dose of patient education assessment pertained to formal group or one-on-one sessions only. Informal and opportunistic patient education, such as that delivered during program intake and orientation, exercise instruction, as well as other routine clinical interactions, would vary from patient to patient as applicable. It was not consistently captured, leading to potential under-estimation of total educational exposure. Furthermore, coverage of content regarding management of all CV risk factors and rehabilitation was not considered (e.g., nutrition, tobacco cessation, return to life roles).

Data were self-reported and unverified, and social desirability bias may have led to over-estimation of adherence to best practices, particularly in countries with few programs where respondents may have feared identification. However, this bias is unlikely to have affected comparisons across income classifications or Audit decades, as all respondents received identical items. Despite the availability of translation tools, variation in interpretation of survey items across languages and cultural contexts may also have influenced responses. Finally, minor differences in item wording and response options between the 2016 and 2025 Audits warrant caution when interpreting decade-level changes.

This 2025 Global CR Audit—the third ever since the first a decade ago and the second during the COVID-19 disruption—revealed strong continuity in comprehensive delivery of core CR components worldwide, alongside meaningful evolution in risk factor assessment, hybrid education, and program resources. Differences by income class were observed for the number of core components offered, risk factors assessed, and available program resources, though programs in lower-resource contexts continue to deliver care that is high quality and likely well aligned with local needs. Together, these results underscore the commitment of the global CR community to quality, comprehensive secondary prevention delivery and highlight the potential for greater impact through ensuring all programs have the resources to deliver fully comprehensive CR—including a robust education component be it in-person or digital—thereby improving cardiovascular health, quality of life, and survival, particularly in regions bearing the highest burden of disease.

## Supporting information

S1 Acknowledgements The ICCPR Global Cardiac Rehabilitation Audit Update Investigators. (DOCX)

S1 Protocol ICCPR 2025 Global Cardiac Rehabilitation Audit Update study protocol. (PDF)

S1 Appendix ICCPR’s Global Audit Update Survey. (PDF)

S1 Table Key CR components delivered, by income class, country with any CR, and audit decade. (DOCX)

S2 Table Most common risk factors assessed in phase II cardiac rehabilitation initial assessment by country income class, country, and globally in 2025 Audit Update. (DOCX)

## Abbreviations

AACVPR: American Association of Cardiovascular and Pulmonary Rehabilitation
AI: artificial intelligence
BACPR: British Association for Cardiovascular Prevention and Rehabilitation
CI: confidence interval
CR: cardiac rehabilitation
CVD: cardiovascular disease
EAPC: European Association of Preventive Cardiology
GLMM: generalized linear mixed models
ICCPR: International Council of Cardiovascular Prevention and Rehabilitation
LMICs: low- and middle-income countries

## References

[1] NCD Countdown 2030 Collaborators. Benchmarking progress in non-communicable diseases: a global analysis of cause-specific mortality from 2001 to 2019. Lancet. 2025;406(10509):1255–82. doi: 10.1016/S0140-6736(25)01388-1 40945529 PMC7618237

[2] Vaduganathan M, Mensah GA, Turco JV, Fuster V, Roth GA. The global burden of cardiovascular diseases and risk: a compass for future health. J Am Coll Cardiol. 2022;80(25):2361–71. 36368511 10.1016/j.jacc.2022.11.005

[3] Mocumbi AO. Cardiovascular health care in low- and middle-income countries. Circulation. 2024;149(8):557–9. doi: 10.1161/CIRCULATIONAHA.123.065717 38377254

[4] Grace SL, Turk-Adawi KI, Contractor A, Atrey A, Campbell NRC, Derman W, et al. Cardiac rehabilitation delivery model for low-resource settings: an International Council of Cardiovascular Prevention and Rehabilitation Consensus Statement. Prog Cardiovasc Dis. 2016;59(3):303–22. doi: 10.1016/j.pcad.2016.08.004 27542575

[5] Taylor RS, Dalal HM, McDonagh STJ. The role of cardiac rehabilitation in improving cardiovascular outcomes. Nat Rev Cardiol. 2022;19(3):180–94. doi: 10.1038/s41569-021-00611-7 34531576 PMC8445013

[6] Zullo MD, Jackson LW, Whalen CC, Dolansky MA. Evaluation of the recommended core components of cardiac rehabilitation practice: an opportunity for quality improvement. J Cardiopulm Rehabil Prev. 2012;32(1):32–40. doi: 10.1097/HCR.0b013e31823be0e2 22193929

[7] Wijesekera Kankanamge S, Gallagher R, Grace SL, Zhang L, Cunich M, Candelaria D. Effectiveness of quality improvement interventions in cardiac rehabilitation on processes and patient outcomes: a systematic review and meta-analysis. Eur Heart J Qual Care Clin Outcomes. 2025;11(8):1460–73. doi: 10.1093/ehjqcco/qcaf067 40674512 PMC12714378

[8] Pesah E, Supervia M, Turk-Adawi K, Grace SL. A review of cardiac rehabilitation delivery around the world. Prog Cardiovasc Dis. 2017;60(2):267–80. doi: 10.1016/j.pcad.2017.08.007 28844588

[9] Manandi D, Hyun K, Candelaria D, Hollings M, Tu Q, Gauci S, et al. A century of cardiac rehabilitation research: bibliometric review of publication history, keyword trends, and citations. NPJ Cardiovasc Health. 2025;2(1):26. doi: 10.1038/s44325-025-00062-w 41776046 PMC12912429

[10] GBD 2023 Demographics Collaborators. Global age-sex-specific all-cause mortality and life expectancy estimates for 204 countries and territories and 660 subnational locations, 1950-2023: a demographic analysis for the Global Burden of Disease Study 2023. Lancet. 2025;406(10513):1731–810. 41092927 10.1016/S0140-6736(25)01330-3 PMC12535839

[11] Brown TM, Pack QR, Aberegg E, Brewer LC, Ford YR, Forman DE, et al. Core components of cardiac rehabilitation programs: 2024 update: a scientific statement from the American Heart Association and the American Association of Cardiovascular and Pulmonary Rehabilitation. Circulation. 2024;150(18):e328–47. doi: 10.1161/CIR.0000000000001289 39315436

[12] Piepoli MF, Corrà U, Adamopoulos S, Benzer W, Bjarnason-Wehrens B, Cupples M, et al. Secondary prevention in the clinical management of patients with cardiovascular diseases. Core components, standards and outcome measures for referral and delivery: a policy statement from the cardiac rehabilitation section of the European Association for Cardiovascular Prevention & Rehabilitation. Eur J Prev Cardiol. 2014;21(6):664–81. 22718797 10.1177/2047487312449597

[13] British Association of Cardiovascular Prevention and Rehabilitation. The BACPR standards and core components for cardiovascular disease prevention and rehabilitation. 4th ed. 2023 [cited 2025 Oct 2]. Available from: https://static1.squarespace.com/static/66cc563eecc7a22020c7da6c/t/66ffa8f20aef5d0b272c6b0e/1728030962905/BACPR+Standards+and+Core+Components+2023.pdf

[14] Vrints C, Andreotti F, Koskinas KC, Rossello X, Adamo M, Ainslie J, et al. 2024 ESC guidelines for the management of chronic coronary syndromes. Eur Heart J. 2024;45(36):3415–537. doi: 10.1093/eurheartj/ehae177 39210710

[15] Cotie LM, Vanzella LM, Pakosh M, Ghisi GL de M. A systematic review of clinical practice guidelines and consensus statements for cardiac rehabilitation delivery: consensus, divergence, and important knowledge gaps. Can J Cardiol. 2024;40(3):330–46. doi: 10.1016/j.cjca.2023.10.016 38376955

[16] Turk-Adawi K, Supervia M, Lopez-Jimenez F, Pesah E, Ding R, Britto RR, et al. Cardiac rehabilitation availability and density around the globe. EClinicalMedicine. 2019;13:31–45. doi: 10.1016/j.eclinm.2019.06.007 31517261 PMC6737209

[17] Supervia M, Turk-Adawi K, Lopez-Jimenez F, Pesah E, Ding R, Britto RR, et al. Nature of cardiac rehabilitation around the globe. EClinicalMedicine. 2019;13:46–56. doi: 10.1016/j.eclinm.2019.06.006 31517262 PMC6733999

[18] Ghisi GL de M, Xu Z, Liu X, Mola A, Gallagher R, Babu AS, et al. Impacts of the COVID-19 pandemic on cardiac rehabilitation delivery around the world. Glob Heart. 2021;16(1):43. doi: 10.5334/gh.939 34211829 PMC8195253

[19] Ghisi GLM, Mampuya WM, Jiandani M, Jiandani M, Martinez J, Cruz Rivero M. Global variations in staffing, accepted indications and dose of phase II cardiac rehabilitation globally: comparisons from ICCPR’s 2025 audit update. Eur J Prev Cardiol. 2025. doi: 10.1093/eurjpc/zwag292 42207983

[20] Babu AS, Heald FH, Contractor A, Ghisi GLM, Buckley J, Mola A. Building capacity in cardiac rehabilitation through the International Council of Cardiovascular Prevention and Rehabilitation’s Cardiac Rehabilitation Foundations Certification (CRFC) program: evaluation of reach, barriers and impact. J Cardiopulm Rehabil Prev. 2022;42(3):178–82. 34840246 10.1097/HCR.0000000000000655

[21] Turk-Adawi KI, Terzic C, Bjarnason-Wehrens B, Grace SL. Cardiac rehabilitation in Canada and Arab countries: comparing availability and program characteristics. BMC Health Serv Res. 2015;15:521. doi: 10.1186/s12913-015-1183-7 26607235 PMC4660793

[22] Lima de Melo Ghisi G, Pesah E, Turk-Adawi K, Supervia M, Lopez Jimenez F, Grace SL. Cardiac rehabilitation models around the globe. J Clin Med. 2018;7(9):260. doi: 10.3390/jcm7090260 30205461 PMC6162832

[23] Chaves G, Turk-Adawi K, Supervia M, Santiago de Araújo Pio C, Abu-Jeish A-H, Mamataz T, et al. Cardiac rehabilitation dose around the world: variation and correlates. Circ Cardiovasc Qual Outcomes. 2020;13(1):e005453. doi: 10.1161/CIRCOUTCOMES.119.005453 31918580

[24] IBM Corp. IBM SPSS Statistics for Windows, Version 31.0. Armonk, NY: IBM Corp; 2023.

[25] World Bank. World Bank country and lending groups [cited 2025 Oct 2]. Available from: https://datahelpdesk.worldbank.org/knowledgebase/articles/906519-world-bank-country-and-lending-groups

[26] Neuendorf KA. The content analysis guidebook. 2nd ed. Thousand Oaks, CA: Sage Publications; 2017.

[27] Pesah E, Turk-Adawi K, Supervia M, Lopez-Jimenez F, Britto R, Ding R, et al. Cardiac rehabilitation delivery in low/middle-income countries. Heart. 2019;105(23):1806–12. doi: 10.1136/heartjnl-2018-314486 31253695

[28] McEvoy JW, Jennings C, Kotseva K, De Bacquer D, De Backer G, Erlund I, et al. Variation in secondary prevention of coronary heart disease: the INTERASPIRE study. Eur Heart J. 2024;45(39):4184–96. doi: 10.1093/eurheartj/ehae558 39212219

[29] van Zyl C, Badenhorst M, Hanekom S, Heine M. Unravelling “low-resource settings”: a systematic scoping review with qualitative content analysis. BMJ Glob Health. 2021;6(6):e005190. doi: 10.1136/bmjgh-2021-005190 34083239 PMC8183220

[30] Shi W, Ghisi GLM, Zhang L, Hyun K, Pakosh M, Gallagher R. Systematic review, meta-analysis and meta-regression to determine the effects of patient education on health behaviour change in adults diagnosed with coronary heart disease. J Clin Nurs. 2023;32(15–16):5300–27. doi: 10.1111/jocn.16519 36088570

[31] Shi W, Ghisi GLM, Zhang L, Hyun K, Pakosh M, Gallagher R. A systematic review, meta-analysis, and meta-regression of patient education for secondary prevention in patients with coronary heart disease: impact on psychological outcomes. Eur J Cardiovasc Nurs. 2022;21(7):643–54. doi: 10.1093/eurjcn/zvac001 35134883

[32] Ghisi GL de M. Transforming patient education in cardiac rehabilitation: a vision for the future. Patient Educ Couns. 2025;138:109176. doi: 10.1016/j.pec.2025.109176 40409014

[33] Kizilkilic SE, Xu L, Akinci B, Brørs G, Bäck M, Baritello O, et al. Educational methods to improve digital health literacy: a systematic review and meta-analysis for the EAPC Opti(MI)se initiative. Eur J Prev Cardiol. 2025;:zwaf354. doi: 10.1093/eurjpc/zwaf354 40659537

[34] Ghisi GL de M, Rouleau F, Ross M-K, Dufour-Doiron M, Belliveau SL, Brideau J-R, et al. Effectiveness of an education intervention among cardiac rehabilitation patients in canada: a multi-site study. CJC Open. 2020;2(4):214–21. doi: 10.1016/j.cjco.2020.02.008 32695971 PMC7365818

[35] Ghisi GL de M, Grace SL, Anchique CV, Gordillo X, Fernandez R, Quesada D, et al. Translation and evaluation of a comprehensive educational program for cardiac rehabilitation patients in Latin America: a multi-national, longitudinal study. Patient Educ Couns. 2021;104(5):1140–8. doi: 10.1016/j.pec.2020.10.008 33097358 PMC7550271

[36] Liu X, Grace SL, Ghisi GLM, Shi W, Shen C, Oh P, et al. Controlled pilot test of a translated cardiac rehabilitation education curriculum in percutaneous coronary intervention patients in a middle-income country delivered using WeChat: acceptability, engagement, satisfaction and preliminary outcomes. Health Educ Res. 2022;37(5):314–32. doi: 10.1093/her/cyac022 36087021

[37] Gray R, Indraratna P, Lovell N, Ooi S-Y. Digital health technology in the prevention of heart failure and coronary artery disease. Cardiovasc Digit Health J. 2022;3(6 Suppl):S9–16. doi: 10.1016/j.cvdhj.2022.09.002 36589760 PMC9795268

[38] Johnson AM, Brimhall AS, Johnson ET, Hodgson J, Didericksen K, Pye J, et al. A systematic review of the effectiveness of patient education through patient portals. JAMIA Open. 2023;6(1):ooac085. doi: 10.1093/jamiaopen/ooac085 36686972 PMC9847535

[39] Williams JAS, Byles JE, Inder KJ. Equity of access to cardiac rehabilitation: the role of system factors. Int J Equity Health. 2010;9:2. doi: 10.1186/1475-9276-9-2 20205776 PMC2823593

