# Supplementary material for: Comparisons of core component delivery in cardiac rehabilitation programs by country income classification and decade based on the 2025 Global Audit Update: A survey study
Source: PLoS Med. 2026 Jun 23;23(6):e1005151. doi: 10.1371/journal.pmed.1005151 (PMC13289909; doi:10.1371/journal.pmed.1005151)
Supplement: S1 Acknowledgements — (DOCX) [file pmed.1005151.s001.docx]

The ICCPR Global Cardiac Rehabilitation Audit Update Investigators:

1. Ladislav Batalik, PhD, CRFC^a^
2. Ssu-Yuan Chen, MD, PhD^b,c^
3. Rosalia Fernández, PhD^d,e^
4. Ngone Diaba Gaye, MD, FWACP, CRFC^f^
5. Lela Maskhulia, MD, PhD^g^
6. Pamela Seron, PhD^h^
7. Basuni Radi, MD^i^
8. Miho Nishitani Yokoyama, MD^j^

**Affiliations:**

**a.** Department of Rehabilitation, University Hospital Brno, Brno, Czech Republic; Rehabilitation Clinic, Masaryk University, Brno, Czech Republic
**b.** Cardiovascular and Pulmonary Rehabilitation Center and Division of Physical Medicine & Rehabilitation, Fu Jen Catholic University Hospital, New Taipei City, Taiwan
**c.** Department of Physical Medicine and Rehabilitation, National Taiwan University Hospital and National Taiwan University College of Medicine, Taipei, Taiwan
**d.** Cardiac Rehabilitation, National Cardiovascular Institute – INCOR, Lima, Perú
**e.** San Fernando Medical School, National University of San Marcos, Lima, Perú
**f.** Cardiac Rehabilitation, Ibra Mamadou Wane Medical Center, Dakar, Senegal
**g.** Physical Medicine, Tbilisi State Medical University, Tbilisi, Georgia
**h.** Department of Rehabilitation Sciences, University of La Frontera, Temuco, Chile
**i.** National Cardiovascular Center Harapan Kita, Indonesia
**j.** Graduate School of Medicine, Juntendo University, Japan
