# Supplementary material for: Comparisons of core component delivery in cardiac rehabilitation programs by country income classification and decade based on the 2025 Global Audit Update: A survey study
Source: PLoS Med. 2026 Jun 23;23(6):e1005151. doi: 10.1371/journal.pmed.1005151 (PMC13289909; doi:10.1371/journal.pmed.1005151)
Supplement: S1 Protocol — (PDF) [file pmed.1005151.s002.pdf]

Study Protocol

Version 3; November 4, 2025

Table of Contents

Title: 3
Investigators: 3
Principal Investigators: 3
Trainee: 3
Background: 3
Cardiac Rehabilitation 3
Evidence-practice gaps 4
Study Objectives 5
METHODS 5
Study Design 5
Procedure 5
Quantitative: 5
Qualitative (OBJ 3): 6
Sample 7
Quantitative: 7
Qualitative (Obj 3): 8
Measures 8
Quantitative: 8
Qualitative (Objective 3): 10
Data Analyses 10
Quantitative: 10
Qualitative/ Objective 3: 11
Ethical Considerations 12
Data Handling / Security 12
Quantitative: 12
Qualitative: 12
Knowledge Translation 13

Budget and Funding 13
Timeline 13
References 14

Title: Cardiac Rehabilitation Access Globally Post-Pandemic: A Mixed-Method Study

Investigators:

Principal Investigators:

Sherry L. Grace, PhD; York University and University Health Network, Toronto Canada
ICCPR Executive Board
sgrace@yorku.ca

Gabriela Ghisi, PhD; University Health Network, Toronto Canada and York University
ICCPR Executive Board
gabriela.ghisi@gmail.com

Trainee: Rachael Carson, PhD Student, York University

Background:

Cardiovascular diseases (CVDs) are the leading cause of mortality globally, claiming over 17 million lives annually.[1] Notably, low- and middle-income countries (LMICs; i.e., based on World Bank classification)[2] have experienced a disconcerting surge in CVD deaths over the past two decades, with more than 80% of world's CVD deaths concentrated in these countries.[1] Projections indicate that CVD is poised to remain the single leading causes of death by 2030.[3] While advancements in medical technology and treatment have led to a decline in CVD mortality rates in high-income countries, the prevalence and incidence remain high.[4] Consequently, the number of individuals grappling with chronic CVDs and associated disabilities is on the rise, contributing to 10% of the global burden of disease.[1,2] CVD is also on the rise as a significant health burden in developing nations, with particular concern related to the comparatively early onset of CVD-related deaths in LMICs in contrast to developed countries,[1] resulting in more pronounced negative impacts on economic productivity and the labor force.[5]

Indeed, the economic impact of CVD on health systems is substantial, encompassing both direct and indirect costs such as expenses related to healthcare services, medications, and lost productivity due to mortality and morbidity.[6,7] CVD costs the European Union €$210 billion a year,[9] and the American economy US$407.3 billion in 2019.[10]

Cardiac Rehabilitation

Cardiac Rehabilitation (CR) is a comprehensive outpatient secondary prevention model of care which mitigates this health and economic burden.[11,12] CR programs deliver individualized, inter-professional care, including: medical assessment, structured exercise training, patient and family education, cardiovascular risk factor management (e.g., optimization of medications to control

lipids, hypertension, tobacco use) and psychosocial counselling.[13] Patients generally come to a clinical centre for 1 hour-long sessions per week over several months.[14] Patients are very satisfied with CR care.[15]

The many clinical benefits of CR are supported by rigorous meta-analyses randomized controlled trials.[16] It is internationally-agreed that CR participation enhances quality of life in a clinically meaningful way,[16-21] and lowers morbidity and mortality by approximately 25%.[19] CR improves return to work and reduces downstream healthcare utilization, at very low cost.[22,23] For these reasons, clinical practice guidelines around the world recommend CR referral for CVD patients.[24]

Evidence-practice gaps

Despite these benefits, unfortunately CR is grossly under-used around the world.[25] When compared to other clinical guideline recommendations for CVD inpatients[26] and outpatients,[27] CR referral is the least well-implemented. Data from the United States (US) shows <30% of CR-indicated patients enrol.[29] Data shows that if every CR program in the US was filled to capacity plus 10%, still only 45% of CR-indicated patients could be served.[30] Except Sweden[31] and the United Kingdom,[32] access rates are also abysmal in the only other countries where data are available, such as Australia,[33] Europe,[34] Japan,[35] and South Korea.[36] Many countries experiencing a demographic transition from communicable to non-communicable diseases are establishing cardiology services with expensive diagnostic and treatment technologies, but neglecting CR despite the Guidelines.[37] In sum, unfortunately CR access is inequitable, with socio-economically disadvantaged patients having even less access,[38] and other vulnerable patients such as those living in rural areas, women, and ethnocultural minorities.[39]

Reasons for CR under-utilization are well-established, with factors from the patient to health system level at play.[25,40] The major issue is lack of available programs to serve patients. Indeed, the International Council of Cardiovascular Prevention and Rehabilitation (ICCPR; PIs on Executive) – an umbrella organization of CR Societies globally- conducted a first Global Audit of CR programs in 2016/2017 which was the first ever to quantify program availability.[41] Of 203 countries recognized by the World Health Organization (WHO), it was established for the first time that only 111 (55%) had any CR; only 17% of countries in Africa have any CR. Annual patient capacity in each program was ascertained and juxtaposed against ischemic heart disease estimate in every country of the world from the Global Burden of Disease (GBD) study revealing only one CR "spot" for every 12 new patients in need each year. Indeed, when summarizing across the globe, countries had a median of only 4 programs, and had capacity to serve a median of only 120 patients per year. Globally, 16.3 million more CR "spots" are needed each year to treat incident ischemic heart disease (IHD) patients, with 14 million of those spots needed in LMIC.[42] For instance, India and China each need over 3 million more spots per year, with Russia 1.2.

ICCPR utilized the results from this first Audit to advocate for increases in CR capacity, with unmet CR need data for each country disseminated widely through national champions.[44] In addition, resources to support development of quality programs, particularly in resource-poor settings, were created.[45-47]

In response to calls from ICCPR members based on reports of massive program closures due to the COVID-19 pandemic, the Audit was repeated in Spring 2020.[48] Disconcertingly, results revealed three-quarters of programs had stopped CR delivery, which would correspond to ~4500 programs affected.[48] For infection prevention and control, many operational programs quickly transitioned to unsupervised delivery, primarily through low-tech modalities such as phones. Hybrid CR is defined as offering patients both on-site and remote/unsupervised delivery. Home-based or unsupervised CR +/- technology allows individuals to engage in CR activities from the comfort of their homes.[59] Unsupervised CR typically involves virtual consultations, the use of online education resources, and technology for exercise monitoring.[60] However, such remote CR was scantly reimbursed by government or insurance companies, raising questions of sustainability and patient access.

Study Objectives

The overall objectives of this mixed-method study are to: (a) update the status of CR availability and capacity around the globe, as well as CR service indicators (shortfall/surplus [n] and coverage [% and rank]), (b) investigate CR delivery mode changes since the pandemic (supervised to unsupervised/hybrid), and (c) understand as well as improve CR access and equity.

METHODS

Study Design

This is a mixed-method study, using an exploratory sequential design[50]. It comprises a cross-sectional Audit with program survey in the first stage. These results will be used to inform the second stage qualitative interviews (please note: degree of breadth to be based on resource availability). The methods will be integrated by means of merging, such that results of both stages will be brought together for analysis contiguously.[51] This will be used to inform knowledge translation (KT).

Procedure

Quantitative:

OBJ 1: Using WHO's list of countries, the initial step of confirming countries with available CR services will involve review of results from the first CR Global Audit[41] and the 2020 CR Audit (i.e., during the pandemic).[48] Verification of CR availability will be undertaken for countries believed to have no CR at that time of the previous Audit or <10 programs.

First, the investigative team will approach contacts for these countries from the previous Audits for information, and where verification is not possible they will: (a) thoroughly search via Google using the term “cardiac rehabilitation” and “name of country”, (b) searching via Google for academic hospitals within these countries, whose websites will then be searched for CR programs, (c) consulting through the ICCPR network for contacts with any other local CR experts, (d) attendance at international conferences organized by CR-related Societies to engage with experts from the respective countries, and (e) seeking contacts and information from other CR-related international organizations (e.g., International Society of Physical and Rehabilitation Medicine [ISPRM]) before any country is designated as having no CR.

Program Identification:

An MS Excel spreadsheet of all countries in the world which offer CR will be generated. Upon identifying countries offering CR, in the countries believed to have at least several programs, an “Audit champion” will be sought. First, we will contact the leadership of the ~50 available CR-related Society through ICCPR’s network requesting a Board member or other appropriate party to serve as champion. In cases where no Society is available, Ministries of Health and international organizations (e.g., ISPRM) will be contacted. If no support can be secured, champions from the previous Audit will be invited.

They will be emailed requesting collaboration in the study in return for potential authorship and provision of country-specific information on CR. Agreeing champions will be asked to circulate the email to participants with the survey link, or to provide us with CR program emails to contact directly ourselves.

Program Survey (OBJ 1-3):

The most responsible clinician of the identified CR programs will be sent an e-mail requesting her/his participation and a link to the survey.

Informed consent will be sought through an online consent form. Respondents will be required to click a box to consent and proceed to complete the survey. The survey will also be administered through a web-based program (REDCap).

Non-respondents will be sent a follow-up e-mail reminder 2 weeks later. Respondents will be provided free access to ICCPR’s clinician training if they elect to receive it, by providing their email at the end of the survey (no other identifying information).

Qualitative (OBJ 3):

Interviews will be held with participants from three stakeholder groups to understand strategies and enablers to CR capacity increases. Champions will be enlisted to approach potential interviewees in specific countries via email and phone. Clinicians volunteering for interview at

the end of the survey will be considered. Through ICCPR also, representatives from Societies in the countries with greatest need will be approached.

Those willing will be extended invitations to participate in one-hour, one-on-one semi-structured interviews via Zoom. After training and piloting, interviews will be conducted by the PhD student; They will not be known to the interviewees. A second team member may be present to record non-verbals, and emotion expressed.

Given English may not be the first language for some interviewees, all interviewees will be provided the questions in advance, they will be shared on screen through the interview, the interviewers will ensure their cameras are on so their faces are visible, and live auto-transcript will be enabled.

Interviews will be digitally audio-recorded. Auto-transcripts will be cleaned verbatim while ensuring anonymity before audio-recording destruction.

Sample

Quantitative:

CR-indicated population (Obj 1): To compute CR need, the most current estimates of annual incidence of IHD will be extracted from the GBD by country.[24] IHD is typically due to narrowing of the coronary arteries, usually due to atherosclerosis. GBD estimates IHD as the aggregate of discrete atherosclerosis sequelae, namely: myocardial infarction, angina, or ischemic cardiomyopathy (heart failure due to IHD). In GBD, for IHD, myocardial infarction is defined as per the Fourth Universal Definition, and coronary artery disease is defined as at least moderate (>50%) stenosis of an epicardial coronary vessel based on angiographic or functional diagnostic testing. Ischemic HF is diagnosed clinically using the Framingham or European Society of Cardiology criteria.[55]

Based on internationally-agreed guidelines (see Table 2 here[24]), IHD and HF are the main diagnoses indicated for CR, thus this represents the number of patients who would be indicated for CR in a given year.[53,54] Note that non-ischemic HF patients are also indicated for CR, but no reliable and comparable source of incidence was available in all countries. Therefore, the estimate of CR need should be considered under. Note evidence suggests patients with valve procedures, rhythm issues, or other vascular conditions (e.g., peripheral arterial disease, stroke) also benefit from CR, but these patients were not considered in the estimate of CR need herein. So again, the CR-indicated population is under-estimated, but is as reliable as available.

CR Program Sample (Obj 1-3):

The population consists of all phase 2 (as defined in North America) CR programs identified in the world. The sample will consist of all identified, consenting programs in the world. The most responsible clinician of the identified CR programs will be asked to complete the survey.

CR Program Inclusion Criteria: Post-acute care CR programs offering at least: (i) initial assessment, (ii) structured aerobic exercise (can be supervised or unsupervised; e.g., remote via technology) and (iii) at least one other component to control CV risk factors (e.g., patient education, psychosocial or dietary counselling, tobacco cessation). Setting can be residential, but is generally outpatient.

Exclusion Criteria: Programs that offer CR solely and temporarily as part of a research study. Phase I and maintenance programs.

Qualitative (Obj 3):

Inclusion: (a) Lead clinicians of existing CR programs, (b) Board members of CR-related societies, and (c) cardiac policy-makers, in countries with the greatest CR service shortfall (e.g., exceeding 250,000 patients per year), lowest CR coverage and/or largest reductions in capacity since the previous Audit will be eligible to participate.

Exclusion: Self-reported lack of proficiency in English, and for countries with CR, staff for whom CR is not in their job purview.

While sample size cannot be determined a priori, we are estimating 30 interviews to reach saturation of themes.[56] When no new information is emerging upon concurrent interview coding, data saturation will be considered achieved.

Measures

Quantitative:

Objective 1:

Countries will be characterized by World Bank income class[2] (i.e., 4) and WHO region (i.e., 6).[57] Surveys without country specified were grouped together separately but maintained in the sample to promote generalizability. The indicators below will be aggregated at the country, region, income class and global analytic levels.

CR need: raw IHD incidence per country from latest Global Burden of Disease estimates (2023; https://vizhub.healthdata.org/gbd-results?params=gbd-api-2021-permalink/6afc006e91ec18286cf82cec24bc38c1), minus correction value as described below.

To conservatively take into account that some patients do not survive an incident event (e.g., 30-day survival) to the point of initiating CR or they suffer comorbidities which render them ineligible for CR (inability to ambulate, comorbid terminal condition, comorbid sensory or cognitive impairment), a correction was applied to national incidence values to represent a more true CR-eligible population estimate. Based on the literature showing inequality in CVD morbidity and mortality,[61] we assumed that 85% of incident IHD patients in HICs would be eligible for CR, 80% in MICs, and 75% in MICs.

CR availability: existence of ≥1 program in a country (yes/no), with the total number of available programs ascertained by the national champions.

CR capacity refers to the number of patients a program reports they could serve annually. For each program, total capacity was computed as the sum of annual supervised and unsupervised capacity. For programs that offered either CR model but had missing or outlier (i.e., >10,000) capacity values, the median capacity for the WHO region of the country in which program is located was imputed. However, while not applicable for supervised models given number of responses, if <5 programs within a region reported capacity for unsupervised CR, the overall median unsupervised program capacity across all MICs was applied (i.e., 75), as none of the remaining countries with missing data were classified as HICs and only 2 responding countries were LICs. Zero capacity reflects no availability.

Median program capacity was determined based on the total capacity values across all programs within that country. This was then multiplied by the number of programs in the country to ascertain national CR capacity.

CR service shortfall(-)/surplus(+): CR capacity (national, regional, income class, and global) minus need (corrected number of incident IHD patients per year by analytic level). During computation of regional and global CR service levels, the capacity for each country was constrained to its estimated need, thereby avoiding redistribution of over-capacity to other countries in which it could not have been operational. Negative values represent a shortfall, and positive values a surplus. Zero would reflect a balance of need and capacity.

CR coverage: is calculated as (capacity / need) × 100%, for each analytic level. During computation of global and regional CR shortfalls, the capacity for each country was constrained to its estimated need, thereby avoiding redistribution of over-capacity to other countries in which it could not have been operational.

Values <100% represent under-capacity; 100% are adequate and >100% potential excess capacity (bearing in mind non-ischemic HF and other indicated patient types could not be considered in CR need). Where capacity is zero, coverage = 0%. Coverage %s were ranked by country (1 best).

<u>Objectives 1-3:</u>

The original survey was developed after collecting all other national CR program surveys and conglomerating items, and then soliciting input from the ICCPR community. The survey was revised from the previously-administered version to address objectives herein, and then soliciting input from the ICCPR community.

The survey assesses: (1) referral and access to the program; (2) program staff, facilities and components offered; (3) characteristics of any supervised model they deliver (e.g., type and

number of patients served, dose), (4) nature of any unsupervised delivery models, and (5) some final questions about quality improvement.

Qualitative (Objective 3):

To characterize the sample of interviewees, prior to the interview, they will be asked to share their: country, occupation (i.e., stakeholder group), sex, and years working in cardiac area.

The semi-structured interview guide has been developed by the PIs; it focuses on barriers and enablers to accessible CR capacity increases. Prior to qualitative data collection, it was piloted in each region/income class, to enable contextual tailoring.

Data Analyses

Quantitative:

Objective 1 (GBD):

Current CR availability and capacity will be characterized descriptively at all analytic levels.

Changes in CR availability (i.e., none versus now have any) from the initial Audit at the country level will be quantified. The rapid COVID Audit did not focus on capacity indicators other then program closure due to the pandemic, and hence change from that Audit were not considered. Change from 2016 to present in percent of countries in each income class and region with any CR availability will be described.

CR capacity was a sum of supervised and unsupervised program capacity; only supervised capacity was considered in the initial Audit. Therefore, capacity changes will only be examined descriptively; for changes, the IHD only data will be used, and inferential tests will not be performed given the improved approach in capturing the indicated sample.

CR service levels and coverage will be computed as outlined above at all analytic levels. Coverage values will be ranked by country.[62]

CR service shortfall/surplus and coverage values incorporate GBD incidence estimates. Their CR-indicated condition category changed from the initial Audit (ACS only to now IHD including also angina and ischemic-caused HF). Given also changes in the capacity measure (inclusion in current Audit of unsupervised capacity, these indicators will be juxtaposed by country descriptively from the first[41] Audit estimates and change computed. Change from the first Audit by income class and region will be described, without inferential tests as above.

Objectives 1-3 (Survey):

Country and program response rates will be computed. SPSS version 31 will be utilized, analyzing all initiated surveys.

The number of responses for each question may vary due to missing data, such as instances where respondents did not answer a question due to legitimate inapplicability or the use of skip logic. For descriptive analyses, percentages will be computed using the number of valid responses for the specific item as the denominator.

Data cleaning was documented in an Audit trail. Given the small CR samples in some countries, except for the CR capacity variable, missing data will not be imputed. For continuous variables, outliers and implausible values were also reviewed and addressed as appropriate.

Descriptive statistics, including frequency with percentage (e.g., mode items) or median with quartiles (e.g., capacity) as appropriate, will be applied to analyze all closed-ended items in the survey. Open-ended items with responses from more than 10% of responding programs will be coded using content analysis.[63]

Mode of CR delivery: A descriptive examination of responding program characteristics will be performed. Delivery mode availability will be described by country, and characteristics of hybrid delivery (e.g., technology, dose, comprehensiveness, multidisciplinarity) by region and income class. Availability (yes/no) of unsupervised delivery specifically, and where yes capacity (patients served per year) and reimbursement (yes/no) will be compared descriptively to the previous Audits at a country level, with current income class, regional and global summary statistics computed. Regional and income class differences (independent variables) in the above parameters of capacity and reimbursement will be tested in an exploratory manner via generalized linear mixed models (GLMM), to account for clustering of programs, where country is the random effect. The models will be fitted via the Poisson distribution log link function.

Objective 3 – Access and equity: Strategies for referral of patients to the CR programs will be described by country, region, and income class, with a focus on eReferral. Barriers to broader delivery will be described in the same manner. Regional and income class differences (independent variables) in referral strategies and barriers (dependent variables) will also be tested each using GLMM as above.

<u>*Qualitative/ Objective 3:*</u>

Braun and Clarke's 6-phased iterative and reflexive thematic content analysis will be employed:[64] 1) becoming familiar with the data, 2) generating codes, 3) generating themes, 4) reviewing themes, 5) defining and naming themes, and 6) locating exemplars.

Trainee and the supervisors will review the transcripts with NVIVO coding software. Initial systematic coding and categorization will be conducted independently through repeated readings and line-by-line analysis. Preliminary themes and subthemes will be generated, and segments

with thematic similarity will be grouped. Consensus will be reached through discussion and debate. Preliminary themes will be shared with interviewees (i.e., member checking). Trustworthiness of findings will be ensured through team consensus, participant confidentiality measures using pseudonym initials, and other best practices to optimize credibility.[65]

Integration of Quantitative and Qualitative Results:

Interpretation of the integrated results will be performed narratively, including consideration of fit of the results from the two stages, with input from the investigative team.

Ethical Considerations

Ethics approval was granted by York University in Toronto, Canada (e2025-057).

This is a minimal risk study. Informed consent will be secured from quantitative and qualitative participants.

Data Handling / Security

Consent forms will be stored separately from completed surveys and interview transcripts, which will each only be identifiable by a research identification number.

Quantitative:

Each completed survey will be assigned a unique numeric research ID through REDCap. Data collected from all CR programs will be entered or imported from REDCap into SPSS, with only the research ID number (any emails provided will be removed).

Electronic survey responses and the MS Excel spreadsheet with GBD incidence/need data as well as capacity, service levels and coverage computations will be stored on a secure server at York University. It will not be stored on any portable media.

The anonymized SPSS file may be shared with champion members who will undertake analysis for the purposes of national / regional manuscript preparation or policy translation. Where the number of programs in a country is few, this will not be shared if programs could be identifiable; programs will be consulted first if investigators are unsure.

Aggregate data only will be reported for policy and academic dissemination.

Qualitative:

No video recordings will be saved. Audio-recordings will be deleted after transcripts are cleaned to be verbatim and anonymized.

The PI will destroy the quantitative and qualitative data 5 years after the final publication from the project.

Knowledge Translation

Participating Societies will receive results summaries specific to their countries for informational and advocacy purposes.

Conference abstract / symposia submission, peer-reviewed manuscripts (including at regional and country-level by collaborating champions), social and formal media engagement will be pursued, among other approaches.

The *model for Large-Scale KT* informs all aspects of the project.[52] KT reach will be evaluated.

Budget and Funding

Funding has been secured from Qatar and York Universities (# IRCC-2025-648).

Timeline

Data collection (qualitative and quantitative 2025
Data analysis 2025-2026
Knowledge translation 2026, 2027

References

1. World Heart Federation. World Heart Observatory, 2023. Available at: https://world-heart-federation.org/world-heart-observatory/ (Accessed on July 23, 2024).

2. World Bank. The world by income and region, 2023. Available at: https://datatopics.worldbank.org/world-development-indicators/the-world-by-income-and-region.html (Accessed on July 23, 2024).

3. Mathers CD, Loncar D. Projections of global mortality and burden of disease from 2002 to 2030. *PLoS Med*. 2006;3(11):e442. doi:10.1371/journal.pmed.0030442

4. Public Health Agency of Canada. Heart Disease in Canada, 2022. Vailable at: https://www.canada.ca/en/public-health/services/publications/diseases-conditions/heart-disease-canada.html (Accessed on July 24, 2024).

5. Bowry AD, Lewey J, Dugani SB, Choudhry NK. The Burden of Cardiovascular Disease in Low- and Middle-Income Countries: Epidemiology and Management. *Can J Cardiol*. 2015;31(9):1151-1159. doi:10.1016/j.cjca.2015.06.028

6. Ekinci G. Economic Impacts of Cardiovascular Diseases: An Econometric Evaluation in Turkey. *Iran J Public Health*. 2023;52(1):118-127. doi:10.18502/ijph.v52i1.11673

7. Tarride JE, Lim M, DesMeules M, et al. A review of the cost of cardiovascular disease. *Can J Cardiol*. 2009;25(6):e195-e202. doi:10.1016/s0828-282x(09)70098-4

8. Conference Board of Canada. Modelling the burden of cardiovascular disease, 2021. Available at: https://www.conferenceboard.ca/focus-areas/health/cardiovascular-disease/ (Accessed on Julhy 23, 2024).

9. European Society of Cardiology. Cardiovascular Disease Statistics, 2019. Available at: https://iris.unibocconi.it/retrieve/handle/11565/4023471/115818/Torbica%20EHJ%202019.pdf (Accessed on July 23, 2024).

10. Tsao CW, Aday AW, Almarzooq ZI, et al. Heart Disease and Stroke Statistics-2023 Update: A Report From the American Heart Association. *Circulation*. 2023;147(8):e93-e621. doi:10.1161/CIR.0000000000001123

11. Taylor RS, Dalal HM, McDonagh STJ. The role of cardiac rehabilitation in improving cardiovascular outcomes. *Nat Rev Cardiol*. 2022;19(3):180-194. doi:10.1038/s41569-021-00611-7

12. Oldridge N, Taylor RS. Cost-effectiveness of exercise therapy in patients with coronary heart disease, chronic heart failure and associated risk factors: A systematic review of economic

evaluations of randomized clinical trials. *Eur J Prev Cardiol*. 2020;27(10):1045-1055. doi:10.1177/2047487319881839

13. Grace SL, Turk-Adawi KI, Contractor A, et al. Cardiac Rehabilitation Delivery Model for Low-Resource Settings: An International Council of Cardiovascular Prevention and Rehabilitation Consensus Statement. *Prog Cardiovasc Dis*. 2016;59(3):303-322. doi:10.1016/j.pcad.2016.08.004

14. Chaves G, Turk-Adawi K, Supervia M, et al. Cardiac Rehabilitation Dose Around the World: Variation and Correlates. *Circ Cardiovasc Qual Outcomes*. 2020;13(1):e005453. doi:10.1161/CIRCOUTCOMES.119.005453

15. Ali S, Chessex C, Bassett-Gunter R, Grace SL. Patient satisfaction with cardiac rehabilitation: association with utilization, functional capacity, and heart-health behaviors. *Patient Prefer Adherence*. 2017;11:821-830. Published 2017 Apr 24. doi:10.2147/PPA.S120464

16. Abraham LN, Sibilitz KL, Berg SK, et al. Exercise-based cardiac rehabilitation for adults after heart valve surgery. *Cochrane Database Syst Rev*. 2021;5(5):CD010876. doi:10.1002/14651858.CD010876.pub3

17. Scherrenberg M, Falter M, Dendale P. Cost-effectiveness of cardiac telerehabilitation in coronary artery disease and heart failure patients: systematic review of randomized controlled trials. *Eur Heart J Digit Health*. 2020;1(1):20-29. doi:10.1093/ehjdh/ztaa005

18. Buckley BJR, de Koning IA, Harrison SL, et al. Exercise-based cardiac rehabilitation vs. percutaneous coronary intervention for chronic coronary syndrome: impact on morbidity and mortality. *Eur J Prev Cardiol*. 2022;29(7):1074-1080. doi:10.1093/eurjpc/zwab191

19. Dibben GO, Faulkner J, Oldridge N, et al. Exercise-based cardiac rehabilitation for coronary heart disease: a meta-analysis. *Eur Heart J*. 2023;44(6):452-469. doi:10.1093/eurheartj/ehac747

20. Taylor RS, Dalal HM, McDonagh STJ. The role of cardiac rehabilitation in improving cardiovascular outcomes. *Nat Rev Cardiol*. 2022;19(3):180-194.doi:10.1038/s41569-02100611-7

21. Williams CA, Wadey C, Pieles G, Stuart G, Taylor RS, Long L. Physical activity interventions for people with congenital heart disease. *Cochrane Database Syst Rev*. 2020;10(10):CD013400. doi:10.1002/14651858.CD013400.pub2

22. Pedersen SM, Kruse M, Zwisler ADO, Helmark C, Pedersen SS, Olsen KR. Return to work: does cardiac rehabilitation make a difference? Danish nationwide register-based study. *Scand J Public Health*. 2023;51(2):179-187. doi:10.1177/14034948211062656

23. Sadeghi M, Rahiminam H, Amerizadeh A, et al. Prevalence of Return to Work in Cardiovascular Patients After Cardiac Rehabilitation: A Systematic Review and Meta-analysis. *Curr Probl Cardiol*. 2022;47(7):100876. doi:10.1016/j.cpcardiol.2021.100876

24. Taylor RS, Fredericks S, Jones I, et al. Global perspectives on heart disease rehabilitation and secondary prevention: a scientific statement from the Association of Cardiovascular Nursing and Allied Professions, European Association of Preventive Cardiology, and International Council of Cardiovascular Prevention and Rehabilitation. *Eur Heart J*. 2023;44(28):2515-2525. doi:10.1093/eurheartj/ehad225

25. Grace SL, Kotseva K, Whooley MA. Cardiac Rehabilitation: Under-Utilized Globally. *Curr Cardiol Rep*. 2021;23(9):118. Published 2021 Jul 16. doi:10.1007/s11886-021-01543-x

26. Harrison RW, Simon D, Miller AL, de Lemos JA, Peterson ED, Wang TY. Association of hospital myocardial infarction volume with adherence to American College of Cardiology/American Heart Association performance measures: Insights from the National Cardiovascular Data Registry. *Am Heart J*. 2016;178:95-101. doi:10.1016/j.ahj.2016.04.002

27. Virani SS, Maddox TM, Chan PS, et al. Provider Type and Quality of Outpatient Cardiovascular Disease Care: Insights From the NCDR PINNACLE Registry. *J Am Coll Cardiol*. 2015;66(16):1803-1812. doi:10.1016/j.jacc.2015.08.017

28. Brady S, Purdham D, Oh P, Grace S. Clinical and sociodemographic correlates of referral for cardiac rehabilitation following cardiac revascularization in Ontario. *Heart Lung*. 2013;42(5):320-325. doi:10.1016/j.hrtlng.2013.07.001

29. Keteyian SJ, Jackson SL, Chang A, et al. Tracking Cardiac Rehabilitation Utilization in Medicare Beneficiaries: 2017 UPDATE. *J Cardiopulm Rehabil Prev*. 2022;42(4):235-245. doi:10.1097/HCR.0000000000000675

30. Wall HK, Stolp H, Wright JS, et al. The Million Hearts Initiative: Catalyzing Utilization of Cardiac Rehabilitation and Accelerating Implementation of New Care Models. *J Cardiopulm Rehabil Prev*. 2020;40(5):290-293. doi:10.1097/HCR.0000000000000547

31. Bäck M, Leosdottir M, Hagström E, et al. The SWEDEHEART secondary prevention and cardiac rehabilitation registry (SWEDEHEART CR registry). *Eur Heart J Qual Care Clin Outcomes*. 2021;7(5):431-437. doi:10.1093/ehjqcco/qcab039

32. British Heart Foundation. National Audit of Cardiac rehab (NACR) Quality and Outcomes Report 2021. https://www.bhf.org.uk/informationsupport/publications/statistics/national-audit-of-cardiac-rehabilitation-quality-and-outcomes-report-2021 (Accessed on July 24, 2024).

33.Tavella R, Kennedy K, Beltrame JF, Spertus J, Rumsfeld J. Characteristics and outcomes of patients undergoing cardiac catheterization procedures in US versus Australian hospitals. Circ Cardiovasc Qual Outcomes. 2014;7:A383.

34. Kotseva K, De Backer G, De Bacquer D, et al. Lifestyle and impact on cardiovascular risk factor control in coronary patients across 27 countries: Results from the European Society of Cardiology ESC-EORP EUROASPIRE V registry. *Eur J Prev Cardiol*. 2019;26(8):824-835. doi:10.1177/2047487318825350

35. Ohtera S, Kato G, Ueshima H, et al. A nationwide survey on participation in cardiac rehabilitation among patients with coronary heart disease using health claims data in Japan. *Sci Rep*. 2021;11(1):20096. doi:10.1038/s41598-021-99516-1

36. Choi HE, Kim C, Lee DJ, Joo JE, Kim HS. Participation and Prognostic Impact of Cardiac Rehabilitation After Acute Coronary Syndrome: Big-Data Study of the Korean National Health Insurance Service. J Korean Med Sci. 2023;38(15):e119. doi: 10.3346/jkms.2023.38.e119

37. Korenfeld Y, Mendoza-Bastidas C, Saavedra L, et al. Current status of cardiac rehabilitation in Latin America and the Caribbean. *Am Heart J*. 2009;158(3):480-487. doi:10.1016/j.ahj.2009.06.020

38. Ragupathi L, Stribling J, Yakunina Y, Fuster V, McLaughlin MA, Vedanthan R. Availability, Use, and Barriers to Cardiac Rehabilitation in LMIC. *Glob Heart*. 2017;12(4):323-334.e10. doi:10.1016/j.gheart.2016.09.004

39. Turk-Adawi K, Ghisi GLM, Grace SL. Availability, Density, and Unmet Need for Cardiac Rehabilitation Around the World. *J Cardiopulm Rehabil Prev*. 2022;42(4):E48-E49. doi:10.1097/HCR.0000000000000715

40. Stewart C, Ghisi GLM, Davis E, Grace SL. Cardiac Rehabilitation Barriers Scale (CRBS). In Krageloh CU, Alyami M, Medvedev ON. (Eds.) *International Handbook of Behavioral Health Assessment,* 2023. Springer, Cham. https://link.springer.com/referenceworkentry/10.1007/978-3-030-89738-3_39-1

41. Turk-Adawi K, Supervia M, Lopez-Jimenez F, et al. Cardiac Rehabilitation Availability and Density around the Globe. *EClinicalMedicine*. 2019;13:31-45. doi:10.1016/j.eclinm.2019.06.007

42. Pesah E, Turk-Adawi K, Supervia M, et al. Cardiac rehabilitation delivery in low/middle-income countries. *Heart*. 2019;105(23):1806-1812. doi:10.1136/heartjnl-2018-314486

43. Tran M, Pesah E, Turk-Adawi K, et al. Cardiac Rehabilitation Availability and Delivery in Canada: How Does It Compare With Other High-Income Countries?. *Can J Cardiol*. 2018;34(10 Suppl 2):S252-S262. doi:10.1016/j.cjca.2018.07.413

44. Babu AS, Lopez-Jimenez F, Thomas RJ, et al. Advocacy for outpatient cardiac rehabilitation globally. *BMC Health Serv Res*. 2016;16:471. doi:10.1186/s12913-016-1658-1

45. Babu AS, Heald FA, Contractor A, et al. Building Capacity Through ICCPR Cardiovascular Rehabilitation Foundations Certification (CRFC): EVALUATION OF REACH, BARRIERS, AND IMPACT. *J Cardiopulm Rehabil Prev*. 2022;42(3):178-182. doi:10.1097/HCR.0000000000000655

46. Chowdhury MI, Turk-Adawi K, Babu AS, et al. Development of the International Cardiac Rehabilitation Registry Including Variable Selection and Definition Process. *Glob Heart*. 2022;17(1):1. doi:10.5334/gh.1091

47. Turk-Adawi KI, Elshaikh U, Contractor A, et al. Development and Evaluation of the International Council of Cardiovascular Prevention and Rehabilitation (ICCPR) Program Certification for Low-Resource Settings. *Int J Gen Med*. 2023;16:5199-5214. doi:10.2147/IJGM.S423209

48. Ghisi GLM, Xu Z, Liu X, et al. Impacts of the COVID-19 Pandemic on Cardiac Rehabilitation Delivery around the World. *Glob Heart*. 2021;16(1):43. doi:10.5334/gh.939

49. Thomas EE, Cartledge S, Murphy B, et al. Expanding access to telehealth in Australian cardiac rehabilitation services: a national survey of barriers, enablers, and uptake. *Eur Heart J Dig Health*. 2023;ztad055 doi: /10.1093/ehjdh/ztad055

50. Fetters MD, Curry LA, Creswell JW. Achieving integration in mixed methods designs-principles and practices. *Health Serv Res*. 2013;48(6 Pt 2):2134-2156. doi:10.1111/1475-6773.12117

51. O'Cathain A, Murphy E, Nicholl J. The quality of mixed methods studies in health services research. *J Health Serv Res Policy*. 2008;13(2):92-98. doi:10.1258/jhsrp.2007.007074

52. Pronovost PJ, Berenholtz SM, Needham DM. Translating evidence into practice: a model for large scale knowledge translation. *BMJ*. 2008;337:a1714. Published 2008 Oct 6. doi:10.1136/bmj.a1714

53. European Society of Cardiology. 2019 Guidelines on Chronic Coronary Syndromes, 2019. https://www.escardio.org/Guidelines/Clinical-Practice-Guidelines/Chronic-Coronary-Syndromes (Accessed on July 23, 2024).

54. Heidenreich PA, Bozkurt B, Aguilar D, et al. 2022 AHA/ACC/HFSA Guideline for the Management of Heart Failure: A Report of the American College of Cardiology/American Heart Association Joint Committee on Clinical Practice Guidelines [published correction appears in Circulation. 2022 May 3;145(18):e1033] [published correction appears in Circulation. 2022 Sep27;146(13):e185] [published correction appears in Circulation. 2023 Apr 4;147(14):e674]. *Circulation*. 2022;145(18):e895-e1032. doi:10.1161/CIR.0000000000001063

55. GBD 2017 Disease and Injury Incidence and Prevalence Collaborators. Global, regional, and national incidence, prevalence, and years lived with disability for 354 diseases and injuries for 195 countries and territories, 1990-2017: a systematic analysis for the Global Burden of Disease Study 2017 [published correction appears in Lancet. 2019 Jun 22;393(10190):e44]. *Lancet*. 2018;392(10159):1789-1858. doi:10.1016/S0140-6736(18)32279-7

56. Saunders B, Sim J, Kingstone T, et al. Saturation in qualitative research: exploring its conceptualization and operationalization. *Qual Quant*. 2018;52(4):1893-1907. doi:10.1007/s11135-017-0574-8

57. World Health Organization. Countries, 2024. Available at: https://data.who.int/countries (Accessed on July 23, 2024).

58. Supervia M, Turk-Adawi K, Lopez-Jimenez F, et al. Nature of Cardiac Rehabilitation Around the Globe. *EClinicalMedicine*. 2019;13:46-56. doi:10.1016/j.eclinm.2019.06.006

59. Thomas RJ, Beatty AL, Beckie TM, et al. Home-Based Cardiac Rehabilitation: A Scientific Statement From the American Association of Cardiovascular and Pulmonary Rehabilitation, the American Heart Association, and the American College of Cardiology. *Circulation*. 2019;140(1):e69-e89. doi:10.1161/CIR.0000000000000663

60. Piepoli MF, Hoes AW, Agewall S, et al. 2016 European Guidelines on cardiovascular disease prevention in clinical practice: The Sixth Joint Task Force of the European Society of Cardiology and Other Societies on Cardiovascular Disease Prevention in Clinical Practice (constituted by representatives of 10 societies and by invited experts). Developed with the special contribution of the European Association for Cardiovascular Prevention & Rehabilitation (EACPR). *Eur Heart J*. 2016;37(29):2315-2381. Doi:10.1093/eurheartj/ehw106

61. GBD 2023 Demographics Collaborators. Global age-sex-specific all-cause mortality and life expectancy estimates for 204 countries and territories and 660 subnational locations, 1950-2023: a demographic analysis for the Global Burden of Disease Study 2023. *Lancet.* 2025;406(10513):1731-1810.

62. Mensah GA, Fuster V, Murray CJL, Roth GA; Global Burden of Cardiovascular Diseases and Risks Collaborators. Global Burden of Cardiovascular Diseases and Risks, 1990-2022. *J Am Coll Cardiol*. 2023;82(25):2350-2473. doi:10.1016/j.jacc.2023.11.007

63. Elo S, Kyngäs H. The qualitative content analysis process. *J Adv Nurs*. 2008;62(1):107-115. doi:10.1111/j.1365-2648.2007.04569.x

64. Clarke V, & Braun, V. *Successful Qualitative Research: A Practical Guide for Beginners*. London, United Kingdom: Sage; 2013.

65. Johnson JL, Adkins D, Chauvin S. A Review of the Quality Indicators of Rigor in Qualitative Research. *Am J Pharm Educ*. 2020;84(1):7120. doi:10.5688/ajpe7120
