# Supplementary material for: Comparisons of core component delivery in cardiac rehabilitation programs by country income classification and decade based on the 2025 Global Audit Update: A survey study
Source: PLoS Med. 2026 Jun 23;23(6):e1005151. doi: 10.1371/journal.pmed.1005151 (PMC13289909; doi:10.1371/journal.pmed.1005151)
Supplement: S1 Appendix — (PDF) [file pmed.1005151.s003.pdf]

CARDIAC REHABILITATION (CR) PROGRAM QUESTIONNAIRE

Instructions:
*you can use Google Translate in your browser to translate the survey to your first language.

Please answer the survey in relation to your **phase II (i.e., post-acute, outpatient) CR program only** (excluding any temporary research-related service capacity). It is preferred a program lead complete the survey if possible.

Answer the questions as applicable by: (1) checking (✓) the appropriate box (sometimes one box and other times you will be asked to check as many boxes as apply), (2) typing in an answer, or (3) entering a number, as indicated. The survey items for which you enter numbers are constrained to one value (i.e., you cannot enter a range. If you would like to enter a range, instead enter the midpoint) and will not accept text. You can report a number to up to 1 decimal place if desired. Enter zero (0) only if the answer is none.

If you would like to go back to complete the survey later: click on the button <*Save & Return later*> that you will see at the bottom of every page of the survey, then:

a. Copy & paste, or write down the validation code that appears. Hit <*close*>
b. Bookmark the survey page in your browser, or enter your email address in the box and the survey link will be sent to you.

Be sure to click the "Submit" button when you reach the end of the survey.

1. What is your Title/Position at the cardiac rehabilitation program? (check ✓all that apply):
   - ☐ Medical Director
   - ☐ Coordinator / Manager / Supervisor
   - ☐ Allied healthcare professional
   - ☐ Nurse
   - ☐ *Other*
     Please specify ____

SECTION A: INFORMATION ABOUT REFERRAL AND ACCESS TO YOUR PHASE II CR PROGRAM

2. In what country is your CR program? ______________

3. City / Region: ___________________(optional)

4. Your CR program is located in an/a:

    - ☐ Urban area (e.g., cities, larger towns)
    - ☐ Non-urban

5. What year did your CR program first open? (Please enter a valid four digit start year)

    _____________ **(year)**

6. Is your phase II CR program located within a hospital?
    - ☐ *Yes*
    - ☐ It is a spa / residential facility
    - ☐ It is located elsewhere **(skip to question 8)**

7. *If yes*, does the hospital have an inpatient cardiology service? (Check one box):
    - ☐ Yes
    - ☐ No (*skip to q8*)

7b. *If yes*, approximately what proportion of your program's annual patients are referred directly from the inpatient hospital setting?

________ **%**

7c. *If yes*, are CR referrals of indicated inpatients tracked at least once annually?
- ☐ More often
- ☐ Yes
- ☐ No

7d. If *yes*, how are these inpatients referred to your cardiac rehab program? (Check all that apply):
- ☐ These patients are referred to our cardiac rehabilitation program **systematically** (e.g., electronic; does not require human memory)
- ☐ These patients are referred to our cardiac rehabilitation program **from inpatient rehab**
- ☐ These patients are **referred** to our cardiac rehabilitation program **before discharge**
- ☐ Patients get an initial phase II cardiac rehab **appointment scheduled before discharge**
- ☐ Someone from **phase II cardiac rehab** speaks to the patients at the bedside about the program
- ☐ A **non-physician provider from the inpatient unit** speaks to the patients at the bedside about the program
- ☐ A **physician** speaks to the patients at the bedside about the program
- ☐ A **peer or volunteer** speaks to the patients at the bedside about the program
- ☐ Patients receive **written materials** about the referral and cardiac rehab and / or are provided a **website** to find out more information (e.g., contact information, directions).
- ☐ I am unsure
- ☐ Other (please specify: ____)

☐ None of the above

7e. Whether you have systematic referral or not, please describe any barriers to systematic referral operation or systematic patient referral:

---

8. Who can refer a patient to your program? (Check all that apply)
   - ☐ Patients can self-refer
   - ☐ Physicians
   - ☐ Allied healthcare providers and / or nurses
   - ☐ Community health care workers
   - ☐ *Other*
     Please specify ___________________________________

9. For patients referred following a cardiac hospitalization, on average how many weeks after discharge does a patient start your program? (i.e., initial assessment appointment)(Please enter a numeric value in the field)

__________ **weeks**

10. Does your program have a waiting list for patients to start the program due to lack of capacity to intake new patients? (please check one box)
   - ☐ *yes*
   - ☐ no
   - ☐ actually we have excess capacity, because not enough patients are referred

10b. If yes, approximately how many patients are currently on the waiting list?

________ **patients**

11. Knowing about any other programs in your region and volumes of patients with conditions indicated for CR at local institutions, how many incident patients in your catchment area do you estimate your program needs to be serving per year so all patients in need can access care?

_____________ **patients per year**

12. Who pays for cardiac rehabilitation? (Check all that apply)
- ☐ Social security / government
- ☐ Hospital or clinical center where the cardiac rehab service is based
- ☐ *Patient* (i.e., out-of-pocket)
- ☐ Private health insurance
- ☐ *Other (e.g., donations, Foundation, grants)*

Please specify ____

12b. *If patients pay*: What is the average **percent** of the total program cost that patients pay, if they complete the program? (Please enter a numeric value only in the field)

_____ %

12c. If patients pay, what percentage of indicated patients do you estimate forego CR due to inability to pay out-of-pocket cost?

_____ %

12d. What is the out-of-pocket cost to patients to participate in all prescribed sessions, if they complete the usual program? (Note: Please enter a numeric value; do not consider supplies, transportation or parking)

____

Amount

12d2. Please specify currency

____

Currency

13. What do you estimate is the cost to your program to serve one (1) patient, if they complete the program? (Note: Please enter a numeric value in the field. If some patients are funded to participate in your program via private funding and some via public, please consider the proportion funded by each model and then take an average. We will take into consideration the funding sources for your program which you reported above. We will assume the currency specified above)

____

Amount

14. Do you do / have any of the following to optimize access for diverse patients? (check all that apply)
    - ☐ Referral materials available in different modalities (e.g., video, written and verbal)
    - ☐ Referral materials available in different languages
    - ☐ Staff that speak different languages or engagement of interpreters where needed
    - ☐ Education materials available in different languages
    - ☐ Written and/or video education materials presented in plain language, for patients of lower literacy and numeracy
    - ☐ Education materials tailored for other vulnerable populations such as women
    - ☐ Nutrition counseling tailored for diversity (e.g., low socioeconomic status, cultural preferences)
    - ☐ Support with transportation is available, or we can offer remote delivery
    - ☐ Peer support for patients identifying with an equity-deserving group

- [ ] *Other strategies to engage equity-deserving groups*
    - Please specify: ___________________________
- [ ] None of above

15. How do you manage patient barriers to participation? (check all that apply)
    - [ ] We discuss barriers with patients routinely
    - [ ] We contact patients who do not show up for sessions as expected
    - [ ] We routinely assess program adherence and completion rates to inform quality improvement initiatives
    - [ ] *We use other strategies to optimize adherence*
        - Please specify: ___________________________
    - [ ] None of above

SECTION B: DETAILS ABOUT YOUR CARDIAC REHABILITATION PROGRAM STAFF, FACILITIES & COMPONENTS

1.Who has overall responsibility for cardiac rehabilitation at your program? (Please check one box)

☐ Cardiologist
☐ Physician specialist in internal medicine
☐ Physical medicine and rehabilitation (physiatrist)
☐ *Physician, other specialty*
If you selected "Physician, other specialty", please specify the specialty here ________________
☐ Nurse
☐ Exercise physiologist / specialist / kinesiologist/ biokineticist
☐ Physiotherapist
☐ *Other*
If you checked "other", please specify the heath profession here _________________________

2. Which types of personnel are part of your cardiovascular rehabilitation (CR) team? If they are part of your team, do they work in CR only, or are they part-time or do they have other department obligations? (Check one box in each row):

	Yes-Fulltime CR	Yes-Partial	No
Cardiologist	☐	☐	☐
Physiatrist (Physical Medicine and Rehabilitation)	☐	☐	☐
Sports Medicine Physician	☐	☐	☐
Other Physician (other than psychiatrist)	☐	☐	☐
Nurse / Nurse-Practitioner	☐	☐	☐
Physiotherapist	☐	☐	☐
Kinesiologist/Exercise Specialist/Biokineticist/Exercise Physiologist	☐	☐	☐
Psychiatrist	☐	☐	☐
Psychologist	☐	☐	☐
Social worker	☐	☐	☐

Dietitian	☐	☐	☐
Pharmacist	☐	☐	☐
Occupational Therapist	☐	☐	☐
Community Health worker	☐	☐	☐
Administrative assistant/ Secretary	☐	☐	☐
Other	☐	☐	☐

Please specify what kind of other physician ___________________________________
Please specify which other type of personnel are part of your team _______________________

3. Do your clinical staff supervising patients during exercise sessions have cardiopulmonary resuscitation (CPR) training / certification?
   - ☐ Yes
   - ☐ Mostly
   - ☐ No

4. Do any staff on your team have CR clinician certification? (check all that apply)
   - ☐ Yes, from ICCPR (i.e., CRFC)
   - ☐ Yes, from AACVPR (i.e., CCRP)
   - ☐ No, just some CR training

5. Is their expertise on your CR team to treat multimordity?
   - ☐ Yes, we can manage patients with comorbidities such as diabetes, lung disease etc
   - ☐ We accept multimorbid patients, but work closely with other specialists to manage these patients
   - ☐ We do not really have the expertise or capacity to address non-cardiac conditions in our program

6. Does your program have each of the following ítems, and if yes, is its' use dedicated to your program or shared with another group (check one option in each row)?

	**Dedicated for CR**	**Shared**	**Not available**
Bicycle ergometer	☐	☐	☐
Treadmill ergometer	☐	☐	☐
Arm cyclo-ergometer	☐	☐	☐
Stress test (no O_2)	☐	☐	☐

Stress test with O_2	☐	☐	☐
Telemetry	☐	☐	☐
Group education room	☐	☐	☐
Gym space	☐	☐	☐
Individual assessment/ Counselling room	☐	☐	☐
Patient change room	☐	☐	☐
Administrative office	☐	☐	☐
Electronic patient charts	☐	☐	☐
Resistance training equipment	☐	☐	☐
Body composition analyzer	☐	☐	☐
Staff meeting room	☐	☐	☐
Staff office space	☐	☐	☐
Other	☐	☐	☐

Please specify what other items your program has ___________________________________

7. In your program, do you assess the following risk factors? (Please check one box per row)

	**Yes**	**No**
Blood pressure	☐	☐
Lipids	☐	☐
Body composition	☐	☐
HbA1c and/or blood glucose	☐	☐
Physical activity	☐	☐
Time spent being sedentary	☐	☐
Poor diet	☐	☐
Tobacco use	☐	☐
Harmful use of alcohol and/or drugs	☐	☐
Sleep apnea	☐	☐
Erectile dysfunction	☐	☐
Depression	☐	☐
Social support	☐	☐
Other factor(s)	☐	☐

Please specify which other factor(s) you assess in your program_______________________

8. Which of the following elements of cardiac rehabilitation are provided in your program? (check one box per row)

	Yes	No	Patient referred elsewhere
a. Initial assessment of cardiac history and risk factors	☐	☐	☐
b. Individual consultation with a physician or nurse	☐	☐	☐
c. Exercise stress test	☐	☐	☐
d. *Other functional capacity test*	☐	☐	☐
e. Assessment of strength (e.g., handgrip)	☐	☐	☐
f. Assessment for comorbities / issues that could impact exercise (e.g., cognition, vision, musculoskeletal / mobility issues, frailty, and / or balance / falls risk)	☐	☐	☐
g. Exercise prescription	☐	☐	☐
h. Supervised exercise training	☐	☐	☐
i. Resistance training	☐	☐	☐
j. Patient education	☐	☐	☐
k. Management of cardiovascular risk factors	☐	☐	☐
l. Prescription and/or titration of secondary prevention medications	☐	☐	☐
m. Nutrition counseling	☐	☐	☐
n. Psychological assessment (e.g., depression)	☐	☐	☐
o. Tobacco cessation intervention and/or counselling	☐	☐	☐
p. Sexual health assessment	☐	☐	☐
q. Vocational counseling / support for return-to-work	☐	☐	☐
r. Stress management / Relaxation techniques	☐	☐	☐
s. End of life counseling	☐	☐	☐
t. Alternative forms of exercise, such as yoga, dance, or tai chi	☐	☐	☐
u. Women-focused classes	☐	☐	☐
v. Inclusion of family / informal caregivers	☐	☐	☐
w. End of program re-assessment	☐	☐	☐
x. Communication of patient assessment results with their primary care provider	☐	☐	☐
y. Follow-up after outpatient program	☐	☐	☐
z. Maintenance program	☐	☐	☐
aa. Other	☐	☐	☐

If applicable, please specify what other functional capacity test is used in your program

____

If applicable, please specify what other alternative forms of exercise are offered in your program

____

If applicable, please specify what components of cardiac rehabilitation are provided in your program

____

9. How many formal group or one-on-one education sessions are provided to each patient in your usual program? (Please enter a numeric value; enter zero if you do not provide education; can include online or in-person, but do not count independent reading of materials by patients)

   _____ **sessions**

10. How many minutes on average is each formal education session? (Please enter a numeric value; enter zero if you do not provide education)

   ______ **minutes**

11. Does your site offer a supervised outpatient Cardiac Rehabilitation program in a clinical setting? (not community-based, which is covered later; and maintenance programs should not be reported)

   ☐ *Yes*
   ☐ No (skip to section D)

SECTION C: Supervised Cardiac Rehabilitation Program

1. How many new cardiac rehabilitation **patients** do you provide service to **each year** in your supervised program? (Please enter a numeric value on patient volume; please report the average number of new patients that start in recent years; if you have a solely home-based model, that will be queried separately so do not include that volume here; do count hybrid patients here)

_________ **patients per year**

2. How many new **patients** do you have capacity to serve **each year**, in terms of staff and space, if they all completed your usual supervised (or hybrid) program? (Please enter a numeric value; please report the number of new *patients that could start your program each year*, not the number of patients you treat each year or the number of sessions you offer; capacity for solely-home based patients is assessed later if applicable, so do not count those patients here)

_____________ **patients per year**

3. Which of the following cardiac diagnoses or indications do you accept for your supervised program? (Check all that apply)

- ☐ Post Myocardial Infarction / acute coronary syndrome
- ☐ Stable coronary artery disease, without a recent event or procedure
- ☐ Post percutaneous coronary intervention (PCI)
- ☐ Post coronary artery bypass graft surgery (CABG)
- ☐ Heart failure with reduced ejection fraction
- ☐ Heart failure with preserved ejection fraction
- ☐ Patients who have had valve surgery/repair or transcatheter aortic valve implantation (TAVI)
- ☐ Heart transplant
- ☐ Patients with ventricular assist devices
- ☐ Arrhythmias (hemodynamically-stable)
- ☐ Patients with implanted devices for rhythm control (i.e., ICD / CRT, pacemaker)
- ☐ Congenital heart disease
- ☐ Cardiomyopathy
- ☐ Abdominal Aortic aneurysm / repair
- ☐ Rheumatic heart disease
- ☐ Patients at high-risk of cardiovascular disease (primary prevention)
- ☐ *Other*
  Please specify ____________________

4. Does your program accept patients without cardiac disease?

- ☐ *Yes*
- ☐ No (skip to question 5)

4b. *If yes*, which of the following non-cardiac primary diagnoses indications do you acceptfor your on-site program? (Check all that apply; we assume many of you treat patients with these comorbidities, but this refers to patients with a primary indication other than CAD)

☐ High-risk primary prevention of CVD
☐ Stroke / transient ischemic attack
☐ Intermittent claudication / peripheral vascular disease
☐ Chronic lung disease
☐ Diabetes
☐ Morbid obesity / bariatric surgery
☐ Cancer
☐ HIV/AIDs
☐ Liver disease
☐ Kidney disease
☐ Dementia / cognitive impairment
☐ *Other*
Please, specify which other non-cardiac diagnosis is accepted in your program_______________

5. How many times in a full program does a typical patient have an individual consult with a physician and/or nurse (Please enter zero [0] if none)?

   ___________ **times**

6. What is the standard <u>duration</u> of the on-site cardiac rehabilitation program that you provide to patients? (Please enter a numeric value.)

   __________ **weeks**

7. On average, for how many sessions does each patient come on-site each week? (i.e., frequency; Note: if you run a residential program, leave this question blank and instead answer the next question; do not report how many sessions your program runs in a week)

   __________ **sessions per week**

7b**.** At your spa/residential program: On average, how many CR sessions do offer patients each day? (Please enter a numeric value in the field or leave blank if not spa/residential)

_____ **sessions / day (residential programs)**

8. On average, how long is each exercise session (including warm up, aerobic exercise, strength training and/ or cool down)? (Please enter a numeric value)

   _______________ **minutes / session**

9. On average, how many patients are in each exercise session? (Please enter a numeric value)

   __________ **patients / session**

10. What is the staff to patient ratio during an average supervised exercise at your program?

Insert here the number of patients per one staff member: ______

11. Which healthcare professional(s) are usually present during exercise sessions? (Check one box in each row)

	Present	Not usually present
Physician	☐	☐
Nurse or nurse-practitioner	☐	☐
Physiotherapist	☐	☐
Kinesiologist/Exercise Specialist/Biokineticist/Exercise Physiologist	☐	☐
Community health worker	☐	☐
Other	☐	☐

Please specify which other healthcare professionals are usually present during exercise sessions: ____

SECTION D- ALTERNATIVE MODELS / SETTINGS OF CARDIAC REHABILITATION DELIVERY

1. Are alternative cardiac rehabilitation models such as home-based or community-based (not maintenance), reimbursable by government or insurance companies in your region?
   - ☐ *Yes*
   - ☐ No

1b. If yes, please specify which outpatient post-discharge models are reimbursable (check all that apply):
- ☐ Synchronous exercise sessions group (video)
- ☐ Synchronous education sessions group (video)
- ☐ 1-1 exercise session with a clinician (video)
- ☐ 1-1 non-exercise appointment with a clinician (audio or video)
- ☐ Asynchronous exercise or education as part of overall program
- ☐ Community-based program
- ☐ *Other*
  - o Please specify: ___________________________________

2. Does your cardiac rehabilitation program offer alternative models / settings of outpatient program delivery than a hospital/clinic-based program? (we are not considering maintenance programs, just outpatient post-discharge programs)

- ☐ *Yes*
- ☐ No

2b. **If yes**, please specify (check all that apply):
- ☐ Home-based (+/- technology)
- ☐ Community-based (i.e., for phase II; this does not refer to a maintenance program)
- ☐ *Hybrid of supervised with home or community-based*
  Please describe the nature of your hybrid model:
  ____
- ☐ *Other*
  Please specify what other alternative model is offered:___________________

3. When did the home-based / hybrid program start? (Please enter a numeric value)

____________ **year**

4. What percentage of your patients are served in a home-based /hybrid program? (Enter 'unknown' if you do not know)(Please enter a numeric value)

____________ **%**

5. How many new cardiac rehabilitation **patients** do you provide service to **each year** in your home-based model? (Please enter a numeric value; please report the number of new patients that start in recent years; DO NOT include patients in a supervised or hybrid model if applicable, as that is counted in section C; this only pertains to outpatient post-acute phase II home-based)

_________ **patients per year**

6. How many new **patients** do you have capacity to serve **each year in the home model**, in terms of staff and space, if they all completed your usual program? (Please enter a numeric value; please report the number of new *patients that could start your program each year*, not the number of patients you treat each year or the number of sessions you offer; DO NOT include patients in a supervised or hybrid model if applicable, as that is counted in section C)

_____________ **patients per year**

7. Do you perceive your program has sufficient capacity to meet need/demand in the home-based / hybrid model?
   ☐ Yes
   ☐ *No*

   7b. *If NO***,** please specify why your program doesn't have sufficient capacity to meet/demand in the home-based /hybrid model (check all that apply):
   ☐ Not fully funded
   ☐ Not enough staff
   ☐ Patients' risk too high for unsupervised exercise
   ☐ Patients don't ask for it
   ☐ Patients don't have the technological or other capacity, so there is no point
   ☐ *Other*
   Please specify the other reason your program doesn't have sufficient capacity in the home-based program____

8. What is the standard duration of the home-based /hybrid cardiac rehabilitation program that you provide to patients? (specify in weeks) (Please enter a numeric value in the field)

__________ **weeks**

9. On average, how many sessions (i.e., formal contact with the Cardiac Rehabilitation staff) does each patient complete in the home-based /hybrid program each month remotely? (frequency; do not report how many sessions your program runs in a month for all home-based patients)

__________ **sessions / month**

10. In your home-based model, how many times are patients asked to come on-site per program on average? (enter zero [0] if none)

__________ **times / program**

11. On what basis are patients offered a home-based /hybrid program? (check all that apply)
   ☐ Risk stratification

- ☐ Patient indication
- ☐ Distance to centre
- ☐ Time or work constraints during the Cardiac Rehabilitation centre hours
- ☐ Transportation barriers
- ☐ Patient choice
- ☐ Cost
- ☐ *Other*
  Please, specify on what other basis are patients offered a home-based program__________

12. Do participants in your home-based /hybrid program receive any materials to support them in the program? (check all that apply)
    - ☐ Yes they receive an activity tracker, or use their phone tracker app (e.g., pedometer, accelerometer, log book)
    - ☐ Yes they receive resistance training materials (e.g., therabands)
    - ☐ Yes they receive education materials (e.g., online)
    - ☐ *Yes they receive other materials*
      Please specify what other materials they receive________________________________
    - ☐ No

13. Which of the following patient levels of cardiac risk do you accept for your home-based program? (Check all that apply)
    - ☐ Low
    - ☐ Moderate
    - ☐ High
    - ☐ Not applicable because we do not risk stratify at our program

14. What forms of communication are used with patients in your home-based program? (check one box per row, to report the frequency)

	Never	Daily	Several Times/week	Weekly	Several times / month	Monthly	Just once
Internet webpage	☐	☐	☐	☐	☐	☐	☐
Email	☐	☐	☐	☐	☐	☐	☐
Webcam / videoconference	☐	☐	☐	☐	☐	☐	☐
Mobile phone call	☐	☐	☐	☐	☐	☐	☐
Smartphone app	☐	☐	☐	☐	☐	☐	☐
Text messages	☐	☐	☐	☐	☐	☐	☐
Log or diary (paper)	☐	☐	☐	☐	☐	☐	☐
Telephone (landline)	☐	☐	☐	☐	☐	☐	☐
In-person / on-site visit	☐	☐	☐	☐	☐	☐	☐
Other	☐	☐	☐	☐	☐	☐	☐

Please specify what other form of communication is used in your home-based program:
____

15. Did you perceive any barriers to using these communication tools?
    - ☐ *Yes*
    - ☐ No

15b. If yes: Check all the barriers that apply:
- ☐ Internet connectivity issues for staff or patients
- ☐ Patient lacks technology access (e.g., patients do not have own device)
- ☐ Patient lacks technological proficiencey
- ☐ Lack of staff technology training and/or support
- ☐ *Other*
  Please specify other perceived barriers to communicating with patients via technology____

16. Which provider types interact directly with the patients in the home-based /hybrid cardiac rehabilitation program? Please check all that apply:
    - ☐ *Physician*
      Please specify the specialty of the physician who interacts directly with the patients in the home-based program: ___________ ________________________________
    - ☐ Nurse
    - ☐ Exercise physiologist /specialist, kinesiologist or the like
    - ☐ Physiotherapist
    - ☐ *Other*
      Please specify who interacts with the patient in the home-based program_____ ___________________ ____________________

17. If you offer supervised and unsupervised models, do patients in the unsupervised model interact less with staff or get a less comprehensive program in any way? (check all that apply)
    - ☐ Not applicable as we don't have both models
    - ☐ It is hybrid, so patients get the same
    - ☐ We do not do as comprehensive an assessment for the home-based model, so they are likely not getting the same care as patients in the supervised model
    - ☐ Patients get the same comprehensiveness of care and multidisciplinary team access in both models
    - ☐ Patients in unsupervised do not receive as many components
    - ☐ Patients in the unsupervised program do not meet with the same variety of healthcare professsionals as patients in the supervised model (e.g., they have a case manager who handles care / liaises as needed)
    - ☐ Patients in the unsupervised program get more 1-1 staff time, but less total time with staff than patients in the supervised model
    - ☐ It depends on the patient's needs and if they want to come in for some specific components
    - ☐ Patients in the home program are advantaged in some ways in terms if comprehensiveness and access to specialists compared to patients in the supervised model
    - ☐ *Other differences*

- Please specify: ______________________________

18. Are patients asked to come on-site for a post-program assessment?
    - ☐ *Yes, always*
    - ☐ *Yes, mostly but in some cases exceptions are made and the post-program assessment is done remotely*
    - ☐ No

    18b. If yes, what proportion of patients do you estimate come in for the post-program assessment?

    ________ %

19. What do you think you would need to be ready and able to significantly increase your program's capacity to provide home-based /hybrid cardiac rehabilitation services to patients?

____
____
____

SECTION E- LAST QUESTIONS ABOUT CR QUALITY IMPROVEMENT

1. Are you familiar with the International Council of Cardiovascular Prevention and Rehabilitation (ICCPR?)
   - ☐ Yes
   - ☐ Somewhat
   - ☐ No

2. Do you assess your program quality in at least one area (e.g., referral, patient outcomes) on a regular basis, and discuss it as a team?
   - ☐ Yes
   - ☐ No, we do not have the capability/capacity for this

3. Is your program interested in certification?
   - ☐ Yes (or we are certified)
   - ☐ If we had human resource capacity to support application only
   - ☐ No

4. Is your site interested in being part of a CR registry?
   - ☐ Yes, or we are already a part
   - ☐ *If we had the support*
   - ☐ *No*

   4b. *If no or "if we had the support"*, why? (check all that apply)
   - ☐ We do not have staff with extra time to get the approvals, trained and started
   - ☐ We do not have staff time to enter data and/or spend extra time with patient assessments
   - ☐ Limited technological proficiency and/or literacy of our patients
   - ☐ Our institution would not be supportive
   - ☐ We would not have capacity to follow-up with patients
   - ☐ *Other*
     - o Please specify: ____

Thank you most sincerely on behalf of the International Council of Cardiovascular Prevention and Rehabilitation for the time and expertise you have committed to complete this important questionnaire.

1. In return for your participation, we would like to offer you (or another member of your team) the opportunity to undertake ICCPR Cardiac Rehabilitation Foundations Certification (CRFC). This is an 8-hour online training, with a module for each core CR component. This education is suitable for clinicians and trainees in the various disciplines involved in delivering cardiac rehab. You would be emailed information on how to access the course for 1 learner, which is a $100USD value. If this is of interest, please provide your email below (which will be stored separately from your survey responses). *Please note, we will not have the opportunity to share information on accessing the training with you until we have finished collecting data from as many programs as possible.*

If you would like to receive CRFC information via email, please check this box:

- ☐ Yes, I would like to receive information to access the CRFC training at no cost, and my email address is:________________________
- ☐ No

2. We are planning to **interview** program leaders in countries with high unmet CR need, regarding what can be done and how to get it done. If applicable to your setting, if you **would be willing to be contacted** to learn more, please provide your email address (will be kept separate from survey responses): ____________________

Finally, if you would like to keep appraised of resources and opportunities in the CR field globally, please subscribe to our low volume email distribution list at: https://globalcardiacrehab.com/ICCPR-News/. We would love to have you as part of our community.

Be sure to click the “Submit” button.
