# Supplementary material for: Comparisons of core component delivery in cardiac rehabilitation programs by country income classification and decade based on the 2025 Global Audit Update: A survey study
Source: PLoS Med. 2026 Jun 23;23(6):e1005151. doi: 10.1371/journal.pmed.1005151 (PMC13289909; doi:10.1371/journal.pmed.1005151)
Supplement: S1 Table — (DOCX) [file pmed.1005151.s004.docx]

S1 Table: Key CR Components Delivered, by Income Class, Country with any CR, and Audit Decade

| **Income Class**  **(mean%±SD)**  Country | **Exercise Training** | | **Management of CV Risk Factors** | | **Nutrition Counselling** | | **Patient Education** | | **Tobacco Cessation Intervention** | | **Psychosocial Management** | |
| --- | --- | --- | --- | --- | --- | --- | --- | --- | --- | --- | --- | --- |
|  | 2016  N=1082 | 2025  N=1505 | 2016  N=1082 | 2025  N=1505 | 2016  N=1082 | 2025  N=1505 | 2016  N=1082 | 2025  N=1505 | 2016  N=1082 | 2025  N=1505 | 2016  N=1082 | 2025  N=1505 |
| **High-income^c^** | **95.9±5.5** | **98.7±4.1** | **98.3±6.0** | **96.1±7.8** | **94.1±7.8** | **80.9±16.9** | **98.1±5.7** | **98.1±6.3** | **80.7±17.9** | **73.7±23.1** | **82.6±22.0** | **76.9±22.6** |
| Australia | 73 (91.3%) | 87 (96.7%) | 77 (98.7%) | 86 (95.6%) | 77 (96.3%) | 72 (80.9%) | 78 (97.5%) | 89 (98.9%) | 51 (64.6%) | 63 (70.8%) | 73 (91.3%) | 77 (85.6%) |
| Austria | 5 (100.0%) | SCS | 5 (100.0%) | SCS | 5 (100.0%) | SCS | 4 (100.0%) | SCS | 5 (100.0%) | SCS | 5 (100.0%) | SCS |
| Bahrain | SP | SP | SP | SP | SP | SP | SP | SP | SP | SP | SP | SP |
| Barbados | SP | SP | SP | SP | SP | SP | SP | SP | SP | SP | SP | SP |
| Belgium | 7 (100.0%) | SCS | 6 (100.0%) | SCS | 7 (100.0%) | SCS | 7 (100.0%) | SCS | 7 (100.0%) | SCS | 7 (100.0%) | SCS |
| Bermuda | SP | SP | SP | SP | SP | SP | SP | SP | SP | SP | SP | SP |
| Brunei Darussalam | SCS | SCS | SCS | SCS | SCS | SCS | SCS | SCS | SCS | SCS | SCS | SCS |
| Canada | 47 (97.9%) | 40 (97.6%) | 47 (97.9%) | 37 (88.1%) | 48 (100.0%) | 33 (78.6%) | 47 (97.9%) | 37 (90.2%) | 33 (68.8%) | 26 (61.9%) | 45 (93.8%) | 25 (59.5%) |
| Chile | SP | 27 (96.4%) | SP | 25 (92.6%) | SP | 10 (40.0%) | SP | 26 (92.9%) | SP | 6 (24.0%) | SP | 8 (30.8%) |
| Costa Rica | 5 (83.3%) | 8 (100.0%) | 6 (100.0%) | 7 (87.5%) | 6 (100.0%) | 5 (62.5%) | 6 (100.0%) | 8 (100.0%) | 4 (66.7%) | 6 (75.0%) | 3 (50.0%) | 8 (100.0%) |
| Croatia | SCS | SP | SCS | SP | SCS | SP | SCS | SP | SCS | SP | SCS | SP |
| Czech Republic | 5 (100.0%) | 9 (100.0%) | 4 (80.0%) | 5 (71.4%) | 5 (100.0%) | 4 (57.1%) | 4 (80.0%) | 7 (87.5%) | 4 (80.0%) | 3 (50.0%) | 3 (60.0%) | 2 (33.3%) |
| Denmark | 5 (100.0%) | SCS | 5 (100.0%) | SCS | 5 (100.0%) | SCS | 5 (100.0%) | SCS | 4 (80.0%) | SCS | 2 (40.0%) | SCS |
| England | 48 (100.0%) | 22 (91.7%) | 45 (95.7%) | 22 (95.7%) | 39 (83.0%) | 20 (87.0%) | - | 24 (100.0%) | 30 (63.8%) | 18 (78.3%) | 38 (80.9%) | 21 (91.3%) |
| Finland | 9 (100.0%) | 4 (100.0%) | 8 (88.9%) | 5 (100.0%) | 9 (100.0%) | 5 (100.0%) | 8 (100.0%) | 4 (100.0%) | 8 (88.9%) | 5 (100.0%) | 9 (100.0%) | 3 (75.0%) |
| France | 11 (91.7%) | 19 (100.0%) | 11 (91.7%) | 19 (100.0%) | 11 (91.7%) | 18 (94.7%) | 12 (100.0%) | 19 (100.0%) | 11 (91.7%) | 18 (94.7%) | 11 (91.7%) | 18 (94.7%) |
| Germany | 27 (96.4%) | 7 (100.0%) | 28 (100.0%) | 7 (100.0%) | 28 (100.0%) | 7 (100.0%) | 25 (100.0%) | 7 (100.0%) | 28 (100.0%) | 7 (100.0%) | 27 (100.0%) | 7 (100.0%) |
| Greece | 4 (100.0%) | 6 (100.0%) | 3 (75.0%) | 5 (100.0%) | 3 (75.0%) | 2 (33.3%) | 3 (75.0%) | 3 (75.0%) | 2 (50.0%) | 0 (0.0%) | 3 (75.0%) | 4 (66.7%) |
| Hungary | 17 (89.5%) | 6 (100.0%) | 19 (100.0%) | 5 (100.0%) | 19 (100.0%) | 6 (100.0%) | 18 (100.0%) | 6 (100.0%) | 17 (89.5%) | 6 (100.0%) | 18 (94.7%) | 6 (100.0%) |
| Ireland | 5 (100.0%) | 33 (100.0%) | 5 (100.0%) | 33 (100.0%) | 5 (100.0%) | 28 (84.8%) | 5 (100.0%) | 33 (100.0%) | 5 (100.0%) | 28 (84.8%) | 5 (100.0%) | 27 (81.8%) |
| Israel | 5 (100.0%) | SCS | 5 (100.0%) | SCS | 5 (100.0%) | SCS | 5 (100.0%) | SCS | 2 (40.0%) | SCS | 2 (40.0%) | SCS |
| Italy | 57 (100.0%) | 7 (87.5%) | 57 (100.0%) | 8 (100.0%) | 52 (91.2%) | 7 (87.5%) | 52 (96.3%) | 7 (87.5%) | 45 (78.9%) | 4 (50.0%) | 44 (77.2%) | 6 (85.7%) |
| Japan | 5 (83.3%) | 41 (100.0%) | 6 (100.0%) | 34 (85.0%) | 6 (100.0%) | 31 (75.6%) | 6 (100.0%) | 38 (95.0%) | 5 (83.3%) | 23 (56.1%) | 4 (66.7%) | 18 (43.9%) |
| Lithuania | 8 (100.0%) | SP | 8 (100.0%) | SP | 6 (75.0%) | SP | 6 (100.0%) | SP | 4 (50.0%) | SP | 8 (100.0%) | SP |
| Luxembourg | - | SP | - | SP | - | SP | - | SP | - | SP | - | SP |
| Malta | SP | SP | SP | SP | SP | SP | SP | SP | SP | SP | SP | SP |
| Netherlands (& Aruba) | 20 (100.0%) | 88 (100.0%) | 20 (100.0%) | 86 (100.0%) | 19 (100.0%) | 81 (92.0%) | 18 (100.0%) | 88 (100.0%) | 15 (78.9%) | 78 (88.6%) | 18 (94.7%) | 87 (98.9%) |
| New Zealand | 21 (87.5%) | 10 (83.3%) | 24 (100.0%) | 13 (100.0%) | 24 (100.0%) | 11 (84.6%) | 15 (100.0%) | 13 (100.0%) | 17 (73.9%) | 5 (38.5%) | 21 (87.5%) | 6 (46.2%) |
| Northern Ireland | 10 (100.0%) | SCS | 10 (100.0%) | SCS | 10 (100.0%) | SCS | 10 (100.0%) | SCS | 10 (100.0%) | SCS | 10 (100.0%) | SCS |
| Norway | - | 10 (100.0%) | - | 10 (100.0%) | - | 10 (100.0%) | - | 10 (100.0%) | - | 9 (90.0%) | - | 7 (70.0%) |
| Panama | SP | SCS | SP | SCS | SP | SCS | SP | SCS | SP | SCS | SP | SCS |
| Poland | 13 (92.9%) | 19 (100.0%) | 14 (100.0%) | 19 (100.0%) | 13 (92.9%) | 19 (100.0%) | 13 (100.0%) | 19 (100.0%) | 11 (78.6%) | 15 (78.9%) | 14 (100.0%) | 20 (100.0%) |
| Portugal | 17 (100.0%) | 4 (100.0%) | 17 (100.0%) | 4 (100.0%) | 17 (100.0%) | 4 (100.0%) | 15 (100.0%) | 4 (100.0%) | 14 (82.4%) | 1 (25.0%) | 12 (70.6%) | 3 (75.0%) |
| Qatar | SP | SP | SP | SP | SP | SP | SP | SP | SP | SP | SP | SP |
| Romania | SCS | SCS | SCS | SCS | SCS | SCS | SCS | SCS | SCS | SCS | SCS | SCS |
| Saudi Arabia | - | SCS | - | SCS | - | SCS | - | SCS | - | SCS | - | SCS |
| Scotland | 17 (89.5%) | 8 (100.0%) | 19 (100.0%) | 8 (100.0%) | 17 (94.4%) | 7 (87.5%) | 19 (100.0%) | 8 (100.0%) | 16 (84.2%) | 7 (87.5%) | 19 (100.0%) | 8 (100.0%) |
| Singapore | 7 (100%) | 5 (100.0%) | 7 (100.0%) | 5 (100.0%) | 7 (100.0%) | 4 (80.0%) | 6 (85.7%) | 5 (100.0%) | 5 (71.4%) | 4 (80.0%) | 4 (57.1%) | 3 (60.0%) |
| Slovak Republic | SP | SP | SP | SP | SP | SP | SP | SP | SP | SP | SP | SP |
| Slovenia | SCS | 5 (100.0%) | SCS | 5 (100.0%) | SCS | 5 (100.0%) | SCS) | 5 (100.0%) | SCS | 5 (100.0%) | SCS | 4 (80.0%) |
| South Korea | 11 (91.7%) | 23 (95.8%) | 12 (100.0%) | 23 (95.8%) | 11 (91.7%) | 17 (73.9%) | 12 (100.0%) | 24 (100.0%) | 10 (83.3%) | 15 (65.2%) | 7 (58.3%) | 10 (43.5%) |
| Spain | 45 (97.8%) | 49 (100.0%) | 46 (100.0%) | 46 (95.8%) | 46 (100.0%) | 41 (85.4%) | 42 (100.0%) | 49 (100.0%) | 41 (89.1%) | 34 (70.8%) | 43 (93.5%) | 39 (81.3%) |
| Sweden | 1 (100.0%) | 21 (100.0%) | 1 (100.0%) | 22 (100.0%) | 1 (100.0%) | 19 (90.5%) | - | 22 (100.0%) | 0 (0.0%) | 19 (86.4%) | 0 (0.0%) | 17 (81.0%) |
| Switzerland | 2 (100.0%) | SP | 2 (100.0%) | SP | 2 (100.0%) | SP | 1 (100.0%) | SP | 2 (100.0%) | SP | 2 (100.0%) | SP |
| Taiwan | 19 (90.5%) | 22 (100.0%) | 21 (100.0%) | 20 (90.9%) | 17 (81.0%) | 16 (72.7%) | 21 (100.0%) | 21 (95.5%) | 19 (90.5%) | 15 (68.2%) | 13 (61.9%) | 7 (31.8%) |
| United Arab Emirates | - | 3 (100.0%) | - | 2 (66.7%) | - | 2 (66.7%) | - | 3 (100.0%) | - | 1 (33.3%) | - | 2 (66.7%) |
| Uruguay | 5 (100.0%) | 5 (100.0%) | 5 (100.0%) | 5 (100.0%) | 5 (100.0%) | 3 (60.0%) | 3 (100.0%) | 4 (100.0%) | 2 (40.0%) | 3 (60.0%) | 3 (60.0%) | 2 (50.0%) |
| United States of America | 56 (98.2%) | 52 (100.0%) | 53 (96.4%) | 49 (94.2%) | 54 (94.7%) | 48 (92.3%) | 47 (100.0%) | 52 (100.0%) | 50 (87.7%) | 48 (92.3%) | 55 (98.2%) | 50 (96.2%) |
| Wales | 14 (93.3%) | SP | 15 (100.0%) | SP | 15 (100.0%) | SP | 14 (100.0%) | SP | 11 (73.3%) | SP | 13 (86.7%) | SP |
| **Upper-middle income^c^** | **89.6±28.3** | **95.5±7.8** | **90.9±27.4** | **95.2±8.9** | **85.2±25.7** | **75.6±16.8** | **96.8±6.3** | **97.1±4.8** | **58.1±29.8** | **61.4±20.8** | **65.3±26.3** | **70.4±23.4** |
| Argentina | SCS | 11 (100.0%) | SCS | 11 (91.7%) | SCS | 10 (83.3%) | SCS | 11 (91.7%) | SCS | 4 (36.4%) | SCS | 5 (41.7%) |
| Azerbaijan | - | 1 (100.0%) | - | 1 (100.0%) | - | 1 (100.0%) | - | 1 (100.0%) | - | - | - | 1 (100.0%) |
| Brazil | 24 (100.0%) | 38 (100.0%) | 20 (83.3%) | 33 (86.8%) | 12 (50.0%) | 17 (45.9%) | 17 (85.0%) | 35 (92.1%) | 6 (25.0%) | 12 (33.3%) | 9 (37.5%) | 11 (30.6%) |
| China | 64 (77.1%) | 204 (90.3%) | 82 (98.8%) | 217 (96.0%) | 76 (91.6%) | 207 (91.6%) | 66 (88.0%) | 221 (97.8%) | 70 (84.3%) | 213 (94.2%) | 64 (78.0%) | 210 (92.9%) |
| Colombia | 43 (91.5%) | 35 (97.2%) | 47 (100.0%) | 35 (97.2%) | 40 (85.1%) | 20 (58.8%) | 47 (100.0%) | 35 (100.0%) | 21 (44.7%) | 17 (51.5%) | 33 (70.2%) | 17 (50.0%) |
| Cuba | 8 (100.0%) | SCS | 8 (100.0%) | SCS | 8 (100.0%) | SCS | 8 (100.0%) | SCS | 4 (50.0%) | SCS | 8 (100.0%) | SCS |
| Dominican Republic | SP | SCS | SP | SCS | SP | SCS | SP | SCS | SP | SCS | SP | SCS |
| Georgia | 7 (100.0%) | 11 (84.6%) | 7 (100.0%) | 13 (100.0%) | 5 (71.4%) | 10 (76.9%) | 5 (100.0%) | 12 (100.0%) | 2 (28.6%) | 6 (50.0%) | 4 (57.1%) | 11 (84.6%) |
| Guatemala | SCS | SCS | SCS | SCS | SCS | SCS | SCS | SCS | SCS | SCS | SCS | SCS |
| Indonesia | 10 (100.0%) | 11 (100.0%) | 10 (100.0%) | 11 (100.0%) | 10 (100.0%) | 6 (54.5%) | 7 (87.5%) | 11 (100.0%) | 10 (100.0%) | 7 (63.6%) | 3 (30.0%) | 3 (27.3%) |
| Iran | 12 (100.0%) | 4 (100.0%) | 12 (100.0%) | 4 (100.0%) | 12 (100.0%) | 3 (75.0%) | 12 (100.0%) | 4 (100.0%) | 7 (63.6%) | 2 (50.0%) | 10 (83.3%) | 3 (75.0%) |
| Jamaica | 1 (100.0%) | SP | 0 (0.0%) | SP | 0 (0.0%) | SP | - | SP | 0 (0.0%) | SP | 0 (0.0%) | SP |
| Kazakhstan | 0 (0.0%) | 7 (100.0%) | 1 (100.0%) | 4 (66.7%) | 1 (100.0%) | 3 (60.0%) | - | 6 (85.7%) | - | 6 (85.7%) | - | 4 (66.7%) |
| Malaysia | 3 (100.0%) | 5 (100.0%) | 3 (100.0%) | 5 (100.0%) | 3 (100.0%) | 5 (100.0%) | 3 (100.0%) | 5 (100.0%) | 3 (100.0%) | 4 (80.0%) | 3 (100.0%) | 5 (100.0%) |
| Mexico | 9 (100.0%) | 59 (98.3%) | 9 (100.0%) | 59 (98.3%) | 9 (100.0%) | 51 (89.5%) | 9 (100.0%) | 57 (96.6%) | 6 (66.7%) | 34 (58.6%) | 7 (77.8%) | 50 (83.3%) |
| Moldova | SP | SP | SP | SP | SP | SP | SP | SP | SP | SP | SP | SP |
| Montenegro | - | SP | - | SP | - | SP | - | SP | - | SP | - | SP |
| Paraguay | SCS | SP | SCS | SP | SCS | SP | SCS) | SP | SCS | SP | SCS | SP |
| Peru | 7 (100.0%) | 8 (100.0%) | 7 (100.0%) | 8 (100.0%) | 6 (85.7%) | 6 (75.0%) | 6 (100.0%) | 8 (100.0%) | 1 (14.3%) | 3 (33.3%) | 5 (71.4%) | 7 (87.5%) |
| Serbia | SCS | 3 (75.0%) | SCS | 4 (100.0%) | SCS | 3 (75.0%) | SCS | 4 (100.0%) | SCS | 2 (50.0%) | SCS | 3 (75.0%) |
| South Africa | 13 (100.0%) | SP | 12 (100.0%) | SP | 9 (69.2%) | SP | 11 (100.0%) | SP | 6 (50.0%) | SP | 7 (58.3%) | SP |
| Suriname | - | SP | - | SP | - | SP | - | SP | - | SP | - | SP |
| Thailand | - | 10 (100.0%) | - | 7 (70.0%) | - | 5 (50.0%) | - | 9 (90.0%) | - | 6 (60.0%) | - | 5 (55.6%) |
| Turkey | 8 (100.0%) | 5 (100.0%) | 8 (100.0%) | 4 (100.0%) | 8 (100.0%) | 3 (75.0%) | 8 (100.0%) | 5 (100.0%) | 5 (62.5%) | 3 (75.0%) | 4 (50.0%) | 2 (50.0%) |
| **Lower-middle income^c^** | **100.0±NA** | **97.8±4.5** | **99.2±2.4** | **94.8±8.1** | **100.0±NA** | **94.8±8.1** | **100.0±NA** | **100.0±NA** | **77.8±20.2** | **70.3±21.1** | **86.7±23.1** | **59.0±25.3** |
| Bangladesh | 1 (100.0%) | SCS | 1 (100.0%) | SCS | 1 (100.0%) | SCS | 1 (100.0%) | SCS | 1 (100.0%) | SCS | 1 (100.0%) | SCS |
| Cameroon | - | SP | - | SP | - | SP | - | SP | - | SP | - | SP |
| Cote d’Ivoire | - | 4 (100.0%) | - | 4 (100.0%) | - | 4 (100.0%) | - | 4 (100.0%) | - | 3 (75.0%) | - | 1 (25.0%) |
| Honduras | SP | SCS | SP | SCS | SP | SCS | SP | SCS | SP | SCS | SP | SCS |
| India | 15 (100.0%) | 61 (96.8%) | 15 (100.0%) | 58 (93.5%) | 15 (100.0%) | 49 (79.0%) | 15 (100.0%) | 62 (100.0%) | 11 (73.3%) | 35 (58.3%) | 15 (100.0%) | 39 (67.2%) |
| Jordan | - | SP |  | SP | - | SP | - | SP | - | SP | - | SP |
| Kenya | SP | 4 (100.0%) | SP | 4 (100.0%) | SP | 3 (100.0%) | SP | 4 (100.0%) | SP | 4 (100.0%) | SP | 2 (100.0%) |
| Mauritania | - | SP | - | SP | - | SP | - | SP | - | SP | - | SP |
| Nigeria | SP | SCS | SP | SCS | SP | SCS | SP | SCS | SP | SCS | SP | SCS |
| Pakistan | SCS | 8 (100.0%) | SCS | 8 (100.0%) | SCS | 7 (87.5%) | SCS) | 8 (100.0%) | SCS | 5 (62.5%) | SCS | 4 (50.0%) |
| Philippines | 10 (100.0%) | 12 (100.0%) | 10 (100.0%) | 12 (100.0%) | 10 (100.0%) | 10 (83.3%) | 10 (100.0%) | 12 (100.0%) | 6 (60.0%) | 5 (41.7%) | 6 (60.0%) | 6 (50.0%) |
| Senegal | - | 8 (100.0%) | - | 8 (100.0%) | - | 8 (100.0%) | - | 8 (100.0%) | - | 6 (75.0%) | - | 2 (25.0%) |
| Sri Lanka | SCS | 7 (87.5%) | SCS | 8 (100.0%) | SCS | 8 (100.0%) | SCS | 8 (100.0%) | SCS | 4 (50.0%) | SCS | 5 (62.5%) |
| Tanzania | - | SCS | - | SCS | - | SCS | - | SCS | - | SCS | - | SCS |
| Tunisia | SP | 4 (100.0%) | SP | 3 (100.0%) | SP | 4 (100.0%) | SP | 4 (100.0%) | SP | 4 (100.0%) | SP | 4 (100.0%) |
| Vietnam | - | SCS | - | SCS | - | SCS | - | SCS | - | SCS | - | SCS |
| Zimbabwe | - | SP | - | SP | - | SP | - | SP | - | SP | - | SP |
| **Low income** | **NA** | **NA** | **NA** | **NA** | **NA** | **NA** | **NA** | **NA** | **NA** | **NA** | **NA** | **NA** |
| Malawi | - | 13 (100.0%) | - | 12 (92.3%) | - | 9 (75.0%) | - | 12 (100.0%) | - | 8 (72.7%) | - | 4 (36.4%) |
| Sudan | - | SP | - | SP | - | SP | - | SP | - | SP | - | SP |
| **Not classified^a^** | **NA** | **NA** | **NA** | **NA** | **NA** | **NA** | **NA** | **NA** | **NA** | **NA** | **NA** | **NA** |
| Venezuela | 8 (100.0%) | 4 (100.0%) | 8 (100.0%) | 4 (100.0%) | 8 (100.0%) | 4 (100.0%) | 8 (100.0%) | 4 (100.0%) | 5 (71.4%) | 4 (100.0%) | 6 (75.0%) | 4 (100.0%) |
| **Global^bc^** | **95.2±5.2** | **97.3±1.6** | **96.1±4.3** | **95.4±0.7** | **93.1±7.4** | **90.2±5.2** | **96.8±1.6** | **99.2±1.5**** | **72.2±9.6** | **83.2±6.2** | **78.2±8.7** | **86.3±9.0** |

CV= cardiovascular; CR, cardiac rehabilitation; NA, not applicable; SD, standard deviation.

- among responses received, information about key CR components delivered was not indicated.

SP: data Suppressed to protect program Privacy (i.e., only 1 program in country).

SCS: data suppressed due to Small Cell Sizes rendering estimates unreliable (≤3 programs responding).

^a^surveys of unknown national origin not shown.

^b^differences should not be over-interpreted given in the 2025 survey a response option of “patient referred elsewhere” was available for each core component, and response option was not available in the 2016 survey.

^c^values represent the mean proportion (%) of programs within each World Bank country income classification [25] and globally reporting provision of each core CR element. Percentages are based on valid responses only. Standard deviations (SDs) are also provided where possible to illustrate variability across programs.
