# Supplementary material for: Comparisons of core component delivery in cardiac rehabilitation programs by country income classification and decade based on the 2025 Global Audit Update: A survey study
Source: PLoS Med. 2026 Jun 23;23(6):e1005151. doi: 10.1371/journal.pmed.1005151 (PMC13289909; doi:10.1371/journal.pmed.1005151)
Supplement: S2 Table — (DOCX) [file pmed.1005151.s005.docx]

S2 Table: Most Common Risk Factors Assessed in Phase II Cardiac Rehabilitation Initial Assessment by Country Income Class, Country, and Globally in 2025 Audit Update

| **Income Class**  **(mean%±SD)**  Country | **Blood pressure** | **Physical activity** | **Tobacco use** | **Poor diet** | **Harmful use of alcohol and/or drugs** | **HbA_1_c and/or blood glucose** | **Lipids** | **Depression** |
| --- | --- | --- | --- | --- | --- | --- | --- | --- |
| **High income^b^** | **98.1±4.7** | **96.7±6.6** | **96.7±6.5** | **91.5±10.6** | **91.3±11.6** | **92.0±10.4** | **90.2±19.6** | **89.1±16.4** |
| Australia | 90 (100.0%) | 90 (100.0%) | 89 (98.9%) | 84 (93.3%) | 84 (95.5%) | 63 (77.8%) | 67 (80.7%) | 89 (98.9%) |
| Austria | SCS | SCS | SCS | SCS | SCS | SCS | SCS | SCS |
| Bahrain | SP | SP | SP | SP | SP | SP | SP | SP |
| Barbados | SP | SP | SP | SP | SP | SP | SP | SP |
| Belgium | SCS | SCS | SCS | SCS | SCS | SCS | SCS | SCS |
| Bermuda | SP | SP | SP | SP | SP | SP | SP | SP |
| Brunei Darussalam | SCS | SCS | SCS | SCS | SCS | SCS | SCS | SCS |
| Canada | 42 (100.0%) | 42 (100.0%) | 40 (95.2%) | 36 (85.7%) | 38 (90.5%) | 37 (88.1%) | 35 (83.3%) | 34 (81.0%) |
| Chile | 29 (100.0%) | 27 (96.4%) | 26 (96.3%) | 21 (77.8%) | 20 (80.0%) | 22 (75.9%) | 17 (60.7%) | 17 (63.0%) |
| Costa Rica | 8 (100.0%) | 8 (100.0%) | 8 (100.0%) | 7 (87.5%) | 8 (100.0%) | 8 (100.0%) | 8 (100.0%) | 8 (100.0%) |
| Croatia | SP | SP | SP | SP | SP | SP | SP | SP |
| Czech Republic | 9 (100.0%) | 7 (77.8%) | 8 (100.0%) | 6 (75.0%) | 5 (83.3%) | 5 (83.3%) | 7 (87.5%) | 4 (57.1%) |
| Denmark | SCS | SCS | SCS | SCS | SCS | SCS | SCS | SCS |
| England | 24 (100.0%) | 24 (100.0%) | 24 (100.0%) | 24 (100.0%) | 23 (100.0%) | 20 (83.3%) | 19 (82.6%) | 24 (100.0%) |
| Finland | 5 (100.0%) | 5 (100.0%) | 5 (100.0%) | 5 (100.0%) | 5 (100.0%) | 5 (100.0%) | 5 (100.0%) | 5 (100.0%) |
| France | 19 (100.0%) | 19 (100.0%) | 19 (100.0%) | 19 (100.0%) | 19 (100.0%) | 19 (100.0%) | 19 (100.0%) | 19 (100.0%) |
| Germany | 7 (100.0%) | 7 (100.0%) | 7 (100.0%) | 6 (85.7%) | 7 (100.0%) | 7 (100.0%) | 7 (100.0%) | 7 (100.0%) |
| Greece | 6 (100.0%) | 5 (83.3%) | 4 (80.0%) | 3 (60.0%) | 3 (60.0%) | 4 (80.0%) | 5 (100.0%) | 5 (83.3%) |
| Hungary | 6 (100.0%) | 6 (100.0%) | 6 (100.0%) | 6 (100.0%) | 5 (83.3%) | 6 (100.0%) | 6 (100.0%) | 6 (100.0%) |
| Ireland | 33 (100.0%) | 33 (100.0%) | 33 (100.0%) | 28 (87.5%) | 30 (93.8%) | 31 (93.9%) | 33 (100.0%) | 30 (90.9%) |
| Israel | SCS | SCS | SCS | SCS | SCS | SCS | SCS | SCS |
| Italy | 8 (100.0% | 8 (100.0%) | 8 (100.0%) | 8 (100.0%) | 7 (87.5%) | 8 (100.0%) | 8 (100.0%) | 7 (87.5%) |
| Japan | 41 (100.0%) | 38 (92.7%) | 41 (100.0%) | 35 (85.4%) | 37 (90.2%) | 38 (92.7%) | 36 (90.0%) | 22 (53.7%) |
| Lithuania | SP | SP | SP | SP | SP | SP | SP | SP |
| Luxembourg | SP | SP | SP | SP | SP | SP | SP | SP |
| Malta | SP | SP | SP | SP | SP | SP | SP | SP |
| Netherlands (& Aruba) | 88 (100.0%) | 86 (100.0%) | 88 (100.0%) | 86 (97.7%) | 88 (100.0%) | 78 (91.8%) | 82 (95.3%) | 87 (98.9%) |
| New Zealand | 12 (92.3%) | 12 (92.3%) | 12 (92.3%) | 12 (92.3%) | 12 (92.3%) | 11 (91.7%) | 11 (91.7%) | 11 (84.6%) |
| Northern Ireland | SCS | SCS | SCS | SCS | SCS | SCS | SCS | SCS |
| Norway | 10 (100.0%) | 10 (100.0%) | 10 (100.0%) | 10 (100.0%) | 9 (90.0%) | 9 (90.0%) | 9 (0.0%) | 10 (100.0) |
| Panama | 2 (100.0%) | 2 (100.0%) | 1 (100.0%) | 2 (100.0%) | 2 (100.0%) | 2 (100.0%) | 2 (100.0%) | 2 (100.0%) |
| Poland | 20 (100.0%) | 20 (100.0%) | 20 (100.0%) | 20 (100.0%) | 17 (85.0%) | 11 (57.9%) | 18 (100.0%) | 18 (94.7%) |
| Portugal | 4 (100.0%) | 4 (100.0%) | 4 (100.0%) | 4 (100.0%) | 4 (100.0%) | 4 (100.0%) | 4 (100.0%) | 4 (100.0%) |
| Qatar | SP | SP | SP | SP | SP | SP | SP | SP |
| Romania | SCS | SCS | SCS | SCS | SCS | SCS | SCS | SCS |
| Saudi Arabia | SCS | SCS | SCS | SCS | SCS | SCS | SCS | SCS |
| Scotland | 8 (100.0%) | 8 (100.0%) | 8 (100.0%) | 8 (100.0%) | 8 (100.0%) | 8 (100.0%) | 8 (100.0%) | 8 (100.0%) |
| Singapore | 5 (100.0%) | 5 (100.0%) | 5 (100.0%) | 5 (100.0%) | 3 (60.0%) | 5 (100.0%) | 3 (60.0%) | 4 (80.0%) |
| Slovak Republic | SP | SP | SP | SP | SP | SP | SP | SP |
| Slovenia | 5 (100.0%) | 5 (100.0%) | 5 (100.0%) | 5 (100.0%) | 5 (100.0%) | 5 (100.0%) | 5 (100.0%) | 5 (100.0%) |
| South Korea | 22 (95.7%) | 24 (100.0%) | 23 (100.0%) | 16 (76.2%) | 21 (91.3%) | 20 (90.9%) | 21 (95.5%) | 14 (63.6%) |
| Spain | 49 (98.0%) | 50 (100.0%) | 50 (100.0%) | 50 (100.0%) | 49 (100.0%) | 48 (96.0%) | 49 (98.0%) | 48 (98.0%) |
| Sweden | 23 (100.0%) | 23 (100.0%) | 23 (100.0%) | 23 (100.0%) | 23 (100.0%) | 23 (100.0%) | 22 (100.0%) | 22 (95.7%) |
| Switzerland | SP | SP | SP | SP | SP | SP | SP | SP |
| Taiwan | 23 (100.0%) | 19 (86.4%) | 21 (95.5%) | 19 (86.4%) | 17 (77.3%) | 20 (90.9%) | 19 (86.4%) | 11 (50.0%) |
| United Arab Emirates | 3 (100.0%) | 3 (100.0%) | 3 (100.0%) | 3 (100.0%) | 3 (100.0%) | 3 (100.0%) | 3 (100.0%) | 3 (100.0%) |
| Uruguay | 4 (80.0%) | 5 (100.0%) | 5 (100.0%) | 4 (100.0%) | 3 (75.0%) | 3 (60.0%) | 4 (80.0%) | 5 (100.0%) |
| United States of America | 52 (100.0%) | 51 (98.1%) | 52 (100.0%) | 52 (100.0%) | 46 (88.5%) | 46 (88.5%) | 43 (84.3%) | 52 (100.0%) |
| Wales | SP | SP | SP | SP | SP | SP | SP | SP |
| **Upper-middle income^b^** | **99.7±0.9** | **99.3±1.1** | **99.1±2.3** | **94.0±8.8** | **89.2±13.6** | **91.1±9.7** | **94.5±9.4** | **89.2±13.1** |
| Argentina | 13 (100.0%) | 12 (100.0%) | 13 (100.0%) | 13 (100.0%) | 10 (83.3%) | 11 (91.7%) | 11 (100.0%) | 11 (84.6%) |
| Azerbaijan | 1 (100.0%) | 1 (100.0%) | 1 (100.0%) | 1 (100.0%) | - | 1 (100.0%) | - | 1 (100.0%) |
| Brazil | 38 (97.4%) | 38 (100.0%) | 38 (97.4%) | 31 (79.5%) | 37 (94.9%) | 26 (70.3%) | 27 (71.1%) | 30 (83.3%) |
| China (not incl. Taiwan) | 224 (99.6%) | 220 (97.3%) | 223 (98.7%) | 220 (97.3%) | 218 (96.5%) | 222 (98.2%) | 224 (99.1%) | 209 (92.5%) |
| Colombia | 36 (100.0%) | 35 (97.2%) | 32 (91.4%) | 30 (85.7%) | 31 (91.2%) | 31 (86.1%) | 31 (86.1%) | 21 (63.6%) |
| Cuba | SCS | SCS | SCS | SCS | SCS | SCS | SCS | SCS |
| Dominican Republic | SCS | SCS | SCS | SCS | SCS | SCS | SCS | SCS |
| Georgia | 13 (100.0%) | 13 (100.0%) | 13 (100.0%) | 13 (100.0%) | 13 (100.0%) | 13 (100.0%) | 13 (100.0%) | 12 (92.3%) |
| Guatemala | SCS | SCS | SCS | SCS | SCS | SCS | SCS | SCS |
| Indonesia | 11 (100.0%) | 11 (100.0%) | 11 (100.0%) | 9 (81.8%) | 7 (63.6%) | 10 (90.9%) | 11 (100.0%) | 7 (63.6%) |
| Iran | 4 (100.0%) | 4 (100.0%) | 4 (100.0%) | 4 (100.0%) | 3 (75.0%) | 4 (100.0%) | 4 (100.0%) | 4 (100.0%) |
| Jamaica | SP | SP | SP | SP | SP | SP | SP | SP |
| Kazakhstan | 7 (100.0%) | 7 (100.0%) | 7 (100.0%) | 7 (100.0%) | 6 (100.0%) | 5 (83.3%) | 8 (100.0%) | 7 (100.0%) |
| Malaysia | 5 (100.0%) | 5 (100.0%) | 5 (100.0%) | 4 (80.0%) | 5 (100.0%) | 4 (80.0%) | 4 (80.0%) | 5 (100.0%) |
| Mexico | 60 (100.0%) | 60 (100.0%) | 57 (96.6%) | 59 (98.3%) | 55 (94.8%) | 59 (98.3%) | 60 (100.0%) | 56 (93.3%) |
| Moldova | SP | SP | SP | SP | SP | SP | SP | SP |
| Montenegro | SP | SP | SP | SP | SP | SP | SP | SP |
| Paraguay | SP | SP | SP | SP | SP | SP | SP | SP |
| Peru | 9 (100.0%) | 9 (100.0%) | 9 (100.0%) | 8 (88.9%) | 9 (100.0%) | 9 (100.0%) | 9 (100.0%) | 9 (100.0%) |
| Serbia | 4 (100.0%) | 4 (100.0%) | 4 (100.0%) | 3 (100.0%) | 4 (100.0%) | 4 (100.0%) | 4 (100.0%) | 2 (66.7%) |
| South Africa | SP | SP | SP | SP | SP | SP | SP | SP |
| Suriname | SP | SP | SP | SP | SP | SP | SP | SP |
| Thailand | 10 (100.0%) | 10 (100.0%) | 9 (100.0%) | 7 (77.8%) | 8 (80.0%) | 8 (80.0%) | 8 (80.0%) | 8 (80.0%) |
| Turkey | 5 (100.0%) | 6 (100.0%) | 6 (100.0%) | 5 (100.0%) | 3 (60.0%) | 5 (100.0%) | 6 (100.0%) | 2 (40.0%) |
| **Lower-middle income^b^** | **100.0±NA** | **99.9±0.4** | **97.9±4.2** | **96.6±7.3** | **93.1±8.4** | **99.4±1.9** | **95.5±10.9** | **85.2±17.0** |
| Bangladesh | SCS | SCS | SCS | SCS | SCS | SCS | SCS | SCS |
| Cameroon | SP | SP | SP | SP | SP | SP | SP | SP |
| Cote d’Ivoire | 4 (100.0%) | 4 (100.0%) | 4 (100.0%) | 3 (75.0%) | 3 (75.0%) | 4 (100.0%) | 4 (100.0%) | 2 (50.0%) |
| Honduras | SCS | SCS | SCS | SCS | SCS | SCS | SCS | SCS |
| India | 64 (100.0%) | 62 (98.4%) | 59 (96.7%) | 60 (98.4%) | 58 (93.5%) | 58 (93.5%) | 54 (87.1%) | 45 (75.0%) |
| Jordan | SP | SP | SP | SP | SP | SP | SP | SP |
| Kenya | 4 (100.0%) | 4 (100.0%) | 4 (100.0%) | 3 (100.0%) | 3 (100.0%) | 4 (100.0%) | 4 (100.0%) | 3 (100.0%) |
| Mauritania | SP | SP | SP | SP | SP | SP | SP | SP |
| Nigeria | 3 (100.0%) | 3 (100.0%) | 3 (100.0%) | 3 (100.0%) | 3 (100.0%) | 2 (100.0%) | 2 (100.0%) | 2 (100.0%) |
| Pakistan | 8 (100.0%) | 8 (100.0%) | 7 (87.5%) | 7 (87.5%) | 7 (87.5%) | 7 (100.0%) | 5 (62.5%) | 6 (85.7%) |
| Philippines | 12 (100.0%) | 12 (100.0%) | 10 (90.9%) | 9 (81.8%) | 9 (81.8%) | 11 (100.0%) | 12 (100.0%) | 9 (81.8%) |
| Senegal | 8 (100.0%) | 8 (100.0%) | 8 (100.0%) | 8 (100.0%) | 6 (85.7%) | 8 (100.0%) | 8 (100.0%) | 4 (57.1%) |
| Sri Lanka | 8 (100.0%) | 8 (100.0%) | 8 (100.0%) | 8 (100.0%) | 8 (100.0%) | 8 (100.0%) | 8 (100.0%) | 7 (87.5%) |
| Tanzania | SCS | SCS | SCS | SCS | SCS | SCS | SCS | SCS |
| Tunisia | 4 (100.0%) | 4 (100.0%) | 4 (100.0%) | 4 (100.0%) | 4 (100.0%) | 4 (100.0%) | 4 (100.0%) | 4 (100.0%) |
| Vietnam | SCS | SCS | SCS | SCS | SCS | SCS | SCS | SCS |
| Zimbabwe | SP | SP | SP | SP | SP | SP | SP | SP |
| **Low income** | **NA** | **NA** | **NA** | **NA** | **NA** | **NA** | **NA** | **NA** |
| Malawi | 14 (100.0%) | 14 (100.0%) | 11 (91.7%) | 10 (83.3%) | 10 (90.9%) | 6 (60.0%) | 1 (11.1%) | 9 (64.3%) |
| Sudan | SP | SP | SP | SP | SP | SP | SP | SP |
| **Not classified^a^** | **NA** | **NA** | **NA** | **NA** | **NA** | **NA** | **NA** | **NA** |
| Venezuela | 4 (100.0%) | 4 (100.0%) | 4 (100.0%) | 4 (100.0%) | 4 (100.0%) | 4 (100.0%) | 4 (100.0%) | 4 (100.0%) |
| **Global^b^** | **99.3±1.0** | **98.6±1.8** | **97.9±1.2** | **94.0±2.6** | **91.2±2.0** | **94.2±4.6** | **93.4±2.8** | **87.8±2.3** |

Acronyms: HbA1c, hemoglobin A1c; NA, not applicable; SD, standard deviation.

- among responses received, information about most common risk factors assessed was not indicated.

SP: data Suppressed to protect program Privacy (i.e., only 1 program in country).

SCS: data suppressed due to Small Cell Sizes rendering estimates unreliable (≤3 programs responding).

^a^surveys of unknown national origin not shown.

^b^values represent the mean proportion (%) of programs within each World Bank country income classification [25] and globally reporting provision of each core CR element. Percentages are based on valid responses only. Standard deviations (SDs) are also provided where possible to illustrate variability across programs.
